# Biogeochemical Traits of a High Latitude South Pacific Ocean Calcareous Nannoplankton Community During the Oligocene

**DOI:** 10.1029/2024PA004946

**Published:** 2024-11-28

**Authors:** Rosie M. Sheward, Jens O. Herrle, Julian Fuchs, Samantha J. Gibbs, Paul R. Bown, Pia M. Eibes

**Affiliations:** ^1^ Institute for Geosciences Goethe‐University Frankfurt Frankfurt Germany; ^2^ Department of Earth Science University College London London UK; ^3^ Insitute of Physical Geography Goethe‐University Frankfurt Frankfurt Germany

**Keywords:** phytoplankton, calcification, PIC:POC, cell size, international ocean discovery program

## Abstract

Marine phytoplankton community composition influences the production and export of biomass and inorganic minerals (such as calcite), contributing to core marine ecosystem processes that drive biogeochemical cycles and support marine life. Here we use morphological and assemblage data sets within a size‐trait model to investigate the mix of cellular biogeochemical traits (size, biomass, calcite) present in high latitude calcareous nannoplankton communities through the Oligocene (ca. 34–26 Ma) to better understand the biogeochemical consequences of past climate variability on this major calcifying phytoplankton group. Our record from IODP Site U1553 in the southwest Pacific reveals that nannoplankton communities were most size diverse during the earliest Oligocene, which we propose is linked to evidence for increased nutrient availability in the region across the Eocene‐Oligocene transition. In addition to driving changes in community size structure, early Oligocene extinctions of the largest *Reticulofenestra* species combined with an increasing dominance of heavily calcified, small‐medium‐sized cells through time also led to an overall increase in community inorganic to organic carbon ratios (PIC:POC) throughout the Oligocene. Crucially, genus‐level cellular PIC:POC diversity meant that abundance was not always the best indicator of which species were the major contributors to community biomass and calcite. As shifts in plankton size structure and calcareous nannoplankton PIC:POC have previously been highlighted as important in biological carbon pump dynamics, our results suggest that changes in community composition that are coupled to changes in community biogeochemical trait diversity have the potential to significantly alter the role of calcareous nannoplankton in marine biogeochemical processes.

## Key Points

- Oligocene calcareous nannoplankton exhibit a diversity of size, cellular organic and inorganic carbon traits
- Earliest Oligocene nannoplankton communities at IODP Site U1553 have greater size richness than mid‐late Oligocene communities
- The most abundant species is not necessarily the major contributor to total community organic or inorganic carbon

## 1 Introduction

Our understanding of past phytoplankton ecosystems is based on the fossil records of groups that producemineralized “shells,” primarily diatoms that produce silica frustules, photosynthetic species of organic‐walled dinoflagellates, and calcareous nannoplankton (including coccolithophores) that produce carbonate plates (coccoliths). The biodiversity and assemblage composition of these phytoplankton and other (zoo)plankton groups such as planktonic foraminifera through time are generally well documented and show clear linkages to changes in regional and global climate (e.g., Bown, 2005; Bown et al., 2004; Falkowski & Oliver, 2007; Lazarus et al., 2014; Lowery et al., 2020; Schmidt et al., 2004). Long‐term trends in biodiversity and assemblage composition have also modified community size structure (how abundance and biomass is distributed across size classes) in the planktonic foraminifera (Schmidt et al., 2004), marine diatoms (Finkel et al., 2005), dinoflagellate cysts (Finkel et al., 2007), and calcareous nannoplankton (inferred from coccolith size; Alvarez et al., 2019; Henderiks & Pagani, 2008; Herrmann & Thierstein, 2012; Suchéras‐Marx & Henderiks, 2014). In these studies, size trends were attributed to a combination of macroevolutionary events (e.g., extinction or speciation of particularly large or small species) and changes in assemblage species richness driven by long‐term development of latitudinal and water column thermal gradients, fluctuating nutrient availability, and changing atmospheric CO_2_ concentrations between the early Paleogene “greenhouse” and the Neogene “icehouse” (Norris et al., 2013). Plankton cell/body size regulates carbon fixation, the uptake of key elements such as nitrogen and phosphorus, and energy flows through marine food webs (e.g., Andersen et al., 2016; Berglund et al., 2007; Fuchs & Franks, 2010; Hillebrand et al., 2022; Taniguchi et al., 2014; Woodward et al., 2005). As such, cell size and plankton community size structure are important and widely used traits for exploring nutrient cycling, the biological carbon pump, and ecological interactions under changing ocean conditions (e.g., Agawin et al., 2000; Falkowski et al., 1998; Guidi et al., 2008, 2016; Marañón et al., 2001; Richardson & Jackson, 2007).

Calcareous nannoplankton are an abundant calcifying phytoplankton group that have played a major role in marine carbonate production (ca. 50% to 90% present‐day carbonate fluxes; e.g., Broecker & Clark, 2009; Milliman, 1993; Ziveri et al., 2023) since the Late Triassic through their production of calcite coccoliths used to form a mineralized cell covering (the coccosphere). Calcareous nannoplankton species exhibit considerable size and morphological diversity (Young et al., 2005). Across extant and Paleogene species (Gafar et al., 2019; Gibbs et al., 2018; Sheward & Poulton, 2024; Villiot et al., 2021), cell size ranges between ca. 3–>40 μm (ca. 14–>34,000 μm^3^ biovolume) and cellular particulate organic carbon (POC, also termed biomass) and particulate inorganic carbon (PIC) content have a 20‐ to 40‐fold range across species. The cellular PIC:POC ratio, a relative measure of calcification (normalized to biomass content) that is widely use in carbon cycle models (e.g., Ridgwell et al., 2009), subsequently ranges from <0.05 to >20 across species (Gibbs et al., 2018; Krumhardt et al., 2017; Villiot et al., 2021). Shifts in calcareous nannoplankton assemblage composition and productivity through time can therefore alter the mix of biogeochemical traits in communities (Alvarez et al., 2019; Bordiga et al., 2015; Gibbs et al., 2018; Suchéras‐Marx & Henderiks, 2014) that may alter the transport of organic carbon and biogenic carbonate to the deep ocean, biological carbon pump efficiency, and trophic interactions (Barton et al., 2013; Cermeño & Figueiras, 2008; Dutkiewicz et al., 2015; Edwards et al., 2013; Juranek et al., 2020; Litchman et al., 2007, 2015; Marañón et al., 2012; Ridgwell et al., 2009; Ward et al., 2014).

Here, we explored the influence of community composition on calcareous nannoplankton community size structure, POC and PIC at a high‐latitude site in the southwest Pacific Ocean (IODP Site U1553, Campbell Plateau) between approx. 34.7–26.5 Ma (latest Eocene to mid‐late Oligocene). We present new assemblage composition and morphological trait data sets (including an intact fossil coccosphere data set) and used a cellular size‐trait model to reconstruct the biogeochemical traits of Oligocene calcareous nannoplankton. First, we investigated whether calcareous nannoplankton community size structure at Site U1553 through the latest Eocene‐Oligocene was impacted by changes in community composition. Next, we assessed the influence of species composition, community size structure, and macroevolutionary coccolith size trends on community PIC:POC through time. The late Eocene and Oligocene climate features a distinct global cooling step across the Eocene‐Oligocene transition (EOT) and the onset of a “coolhouse” background state characterized by intervals of warmer and cooler conditions (e.g., Hutchinson et al., 2021; Lear et al., 2008; Liebrand et al., 2017; Liu et al., 2009; O’Brien et al., 2020). Concurrently, the marine environment underwent substantial restructuring (e.g., latitudinal and vertical temperature gradients, nutrient availability) between the mid‐Eocene and late Oligocene that led to widespread reorganization of plankton communities (e.g., Houben et al., 2013; Norris et al., 2013). Our results provide evidence that Eocene‐Oligocene changes in high latitude southwest Pacific ocean conditions influenced calcareous nannoplankton community composition, driving long‐term trends in calcareous nannoplankton community size structure and PIC:POC.

## 2 Materials and Methods

### 2.1 Site Description, Lithology and Age Model

IODP Expedition 378 Site U1553 (52º13.4’ S; 166° 11.5’ E; present‐day water depth 1,221 m) is located in the high latitude South Pacific Ocean, situated south of New Zealand on the western margin of Campbell Plateau (Figure S1 in Supporting Information S1). At its present location, oceanographic conditions are influenced by the position of the Subtropical Front (STF) to the north (located at ca. 42–47° S as it tracks south of South Island) and the Subantarctic Front (SAF) to the south of the Campbell Plateau (ca. 50–55° S). During the late Eocene and Oligocene, Site U1553 is estimated to have been located at approximately 51–55 ºS between 34 and 23 Ma (estimated using paleolatitude.org v3.beta.3, Van Hinsbergen et al., 2015, using the paleomagnetic reference frame of Vaes et al., 2023).

IODP Site U1553 (Holes A–E) recovered 581 m of Cenozoic sediments comprising a thin surface unit of Pleistocene sediments lying disconformably over approx. 200 m of Oligocene‐age nannofossil oozes with varying abundances of foraminifera and radiolarian (Röhl et al., 2022). The Eocene and Oligocene sediments average 90% calcium carbonate in the uppermost 420 m (Röhl et al., 2022). The shallow paleodepth (ca. 1,500 m) and very high carbonate content indicate a depositional environment above the lysocline for the duration of our record (Hollis et al., 1997; Kennett et al., 1975; Pascher et al., 2015). For the age model, we updated the mid‐point depth of six calcareous nannoplankton datums presented in the shipboard age model (Röhl et al., 2022) based on species occurrences in our samples (Text S1; Table S1 in Supporting Information S1). Samples ages were obtained by assuming a linear sedimentation rate between the age‐depth tie points (Geologic Time Scale 2012; GTS2012; Gradstein et al., 2012) shown in Table S1 of Supporting Information S1.

### 2.2 Calcareous Nannoplankton Community Composition

Assemblage composition was investigated in 90 samples spanning the latest Eocene and mid‐late Oligocene (5.505 m‐CCSF to 206.205 m‐CCSF on the data splice; Drury et al., 2022; Röhl et al., 2022) with an average sampling resolution of 2.25 m. Samples for microscopy analysis were prepared using the spraying method of Bollmann et al. (1999) (without the addition of beads), ensuring a thin and random but even distribution of coccoliths on the surface of a glass slide (Blaj & Henderiks, 2007; Henderiks & Törner, 2006). Relative species abundance was quantified using polarized light microscopy (Zeiss Axio Imager.A2) at 1000X magnification (Zeiss EC Plan‐NEOFLUOR 100X/1.3 oil pol objective) with an optical resolution of 0.26 μm. Following a random transect across each slide, a minimum of 300 coccoliths (minimum of 10 fields of view) were identified to species level following the taxonomy detailed on Nannotax3 (Young et al., 2024) or to genus level if diagnostic morphological characteristics were obscured or damaged. The 95% confidence intervals of the relative abundance data were calculated in PAST4 (Hammer et al., 2001) following Suchéras‐Marx et al. (2019).

To assess the (dis)similarity of community composition through time, we performed a non‐metric multidimensional scaling (NMDS) analysis in R (version 4.3.1) based on Bray‐Curtis dissimilarity of the relative abundance of each morphogroup in each sample (*n* = 90) using the metaMDS function in the R package vegan (Oksanen et al., 2024). The number of dimensions were determined by assessing stress values against the dimensions using the dimcheckMDS function (trymax = 5,000; threshold for good fit stress values was <0.2). For visualization, assemblages were grouped a priori based on visual analysis of trends in community composition through the record: Assemblage 1 (latest Eocene and earliest Oligocene, 188 to 206 m‐CCSF), Assemblage 2 (early Oligocene interval of *Clausicoccus* acme, 186 to 164 m‐CCSF), Assemblage 3 (early Oligocene, 116 to 161), Assemblage 4 (mid‐late Oligocene, 18 to 113 m‐CCSF), Assemblage 5 (late Oligocene, 6 to 16 m‐CCSF). To test for the visually observed dissimilarities between the five assemblages, we performed an analysis of variance (ANOSIM, permutations = 9,999). To extract a semi‐quantitative, assemblage‐based indicator of fluctuating temperature and nutrient conditions at Site U1553, we calculated a simple index of the percentage abundance of cold and meso‐eutrophic affinity taxa (*Chiasmoltihus*, *Reticulofenesta daviesii*, *Clausicoccus subdistichus*) and eutrophic affinity taxa (*Chiasmolithus*, *Cl*. *subdistichus*, *Ismolithus recurvus*, *Reticulofenestra lockeri* Gr., *Reticulofenestra umbilicus* Gr.) through the record based on published paleoecological affinities for Paleogene taxa (Persico & Villa, 2004; Viganò et al., 2023; Villa et al., 2008, 2014).

### 2.3 Size and Biogeochemical Trait Modeling

Cellular‐ and community‐level size, POC and PIC traits were reconstructed for 18 snapshots through our latest Eocene to mid‐late Oligocene record (average resolution 480 kyrs). For the purpose of this study, we restricted our analysis of “community” biogeochemical traits to the reconstructed size, POC and PIC traits of common Paleogene genera/species: *Chiasmolithus*, *Cl*. *subdistichus*, *Coccolithus*, *Cyclicargolithus*, *Reticulofenestra*, *Sphenolithus*, *Discoaster*, and *Zygrhablithus bijugatus*. *Reticulofenestra* was further sub‐divided into three morphogroups following Nannotax3 (Young et al., 2024), reflecting different degrees of central area calcification: *Reticulofenestra bisecta* group (*R*. *bisecta*, *R*. *filewiczii*, *R*. *stavensis*), *Reticulofenestra lockeri* group (*R*. *daviesii*, *R*. *gartneri*, *R*. *lockeri*, *R*. *macmillanii*, *R*. *martini*), *Reticulofenestra umbilicus* group (*R*. *umbilicus*, *R*. *dictyoda*, *R*. *hillae*, *R*. *hampdenensis*, *R*. *moorei*, *R*. *circus*, *R*. *oamaruensis*). Collectively, these 10 “morphogroups” comprised >93% of the assemblage at Site U1553 through our record.

We used an existing, data‐driven model (Gibbs et al., 2018) to model community cell size structure at each snapshot based on morphometric and assemblage data sets. We then further developed the original model to reconstruct the distribution of cellular POC and PIC across size classes. All steps of the size‐trait model were implemented in Microsoft Excel using the equations and data sets described in the following sections. A detailed step‐by‐step protocol for the size‐trait model can be found in Gibbs et al. (2018) and is summarized in Text S2 and Figure S6 of Supporting Information S1.

#### 2.3.1 Cell Size Model

Coccosphere morphometric data sets (coccolith length, number of coccoliths per cell, cell size, and coccosphere size measured on individual fossil or extant coccospheres; Gibbs et al., 2013, 2018; Henderiks, 2008; Sheward et al., 2017; see also definitions in Table 1) reveal a robust linear relationship between calcareous nannoplankton cell size (Θ) and coccolith size (*C*_*L*_), first quantified by Henderiks and Pagani (2007) for Neogene Noelaerhabdaceae. Several publications have subsequently estimated temporal changes in cell size scaled to records of coccolith size (e.g., Bordiga et al., 2015; Guitián, Jones, et al., 2020; Henderiks & Pagani, 2007, 2008). The spread of data around the linear relationship between *C*_*L*_ and Θ is systematically related to the number of coccoliths per cell (*C*_*N*_) (e.g., Gibbs et al., 2013, 2018). For any given cell size, the coccosphere can be composed of many very small coccoliths, a few large coccoliths or any intermediate combination of *C*_*L*_ and *C*_*N*_ between these two end‐members.

**Table 1:** Summary of the Terminology, Morphometric and Biogeochemical Parameters as Defined in This Study

	Symbol	Unit	Definition
Terminology:			
Calcareous nannoplankton	n/a	n/a	Plankton ca. 2–63 μm in diameter with a calcareous (CaCO_3_) cell covering. Includes coccolithophores (division Haptophyta) but excludes fragments and juveniles of larger fossils (Young et al., 1997).
Assemblage	n/a	n/a	All calcareous nannoplankton species present at the site at a specific timepoint.
Community	n/a	n/a	Here, calcareous nannoplankton within the 10 morphogroups modeled in this study. For the purpose of our analysis, “community” refers to a fixed standing stock of 100 cells per unit volume seawater.
Morphogroup	n/a	n/a	Informal groupings of species based on similar (coccolith) morphological features. Generally follows genus‐level classifications except for *Reticulofenestra*, which is subdivided into three morphological groups based on coccolith morphology (central area structure, degree of calcification).
Morphometric parameters:			
Coccolith length	*C*_*L*_	μm	Maximum dimension of the coccolith distal (facing outwards from cell surface) shield in the longitudinal direction, unless otherwise defined in the Methods.
Number of coccoliths/cell	*C*_*N*_	n/a	Number of coccoliths forming the coccosphere (calcite cell covering).
Coccosphere diameter	∅	μm	Diameter of the coccosphere (calcite cell covering).
Cell diameter	Θ	μm	Diameter of the organic cell, here measured/modeled as the internal diameter of the coccosphere.
Biogeochemical trait parameters:			
Particulate organic carbon	POC	pg C	Particulate organic carbon content of the cell or all the cells in the community.
Particulate inorganic carbon	PIC	pg C	Particulate inorganic carbon content of the coccosphere or all the cells in the community.
Total community POC	n/a	pg C	Here, the total mass of POC represented by the number of cells of each morphogroup in the community of 10 morphogroups (sample specific), assuming a fixed standing stock of 100 cells.
Biomass	n/a	pg C	An alternative term for the mass of organic carbon per cell or per unit volume seawater.
Total community PIC	n/a	pg C	Here, the total mass of PIC represented by the number of cells of each morphogroup in the community of 10 morphogroups (sample specific), assuming a fixed standing stock of 100 cells.
Particulate inorganic to organic carbon ratio	PIC:POC	mol:mol	The molar ratio between PIC and POC content, either per cell or for the whole calcareous nannoplankton community (total community PIC divided by total community POC). At the community level, this parameter is independent of standing stocks (e.g., cells mL^−1^). A simplified but widely used measure for the export ratio of biogenic carbonate and biomass produced in the surface ocean and an important term in marine carbon cycle models (e.g., Ridgwell et al., 2009).

Gibbs et al. (2018) were the first to model Θ as a function of the taxon‐specific frequency distribution of *C*_*L*_ in a specific sample and the taxon‐specific range of *C*_*N*_ observed in fossil coccospheres. Their cell size model takes the form of a power‐law relationship between *C*_*L*_ and the ratio of *C*_*N*_ to cell surface area (a function of cell radius, from which Θ can be back‐calculated). Here, we followed the approach of Gibbs et al. (2018) to model sample‐specific cell size distributions for placolith‐type genera for which we have fossil coccosphere morphometric data (*Chiasmolithus*, *Clausicoccus*, *Coccolithus*, *Cyclicargolithus*, *Reticulofenestra)*. An overview of this method is visualized in Figure S6 of Supporting Information S1 and is summarized in the following and Text S2 of Supporting Information S1. The original method of Gibbs et al. (2018) was slightly adjusted by parameterizing the Θ–*C*_*L*_–*C*_*N*_ relationship as a log_10_‐transformed linear regression between *C*_*L*_ (*y*‐axis) and cell surface area/*C*_*N*_ (*x*‐axis). The cell size of *C*_*L*_ and *C*_*N*_ combinations was calculated for each morphogroup by rearranging of the line of best fit equation of the relationship between log_10_(*C*_*L*_) and log_10_ (coccolith surface area/*C*_*N*_), following Equation 1:

(1) $$\Theta =2\times \sqrt[{2}]{\frac{\left(C_{N\;}\times \sqrt[{\alpha }]{\frac{C_{L}}{10^{\beta }}}\right)}{4\pi }}$$

where Θ is cell diameter (μm), *C*_*N*_ is number of coccoliths per cell, *C*_*L*_ is coccolith length (μm), and α and β are the slope and *y*‐intercept of the log‐log linear regression, respectively (Figure S3 in Supporting Information S1).

Each species has a realistic minimum to maximum *C*_*L*_ and *C*_*N*_ range. Some combinations of *C*_*L*_ and *C*_*N*_ occur more frequently than others and *C*_*L*_ range may vary through time due to evolution and/or environment. The final frequency distribution of modeled morphogroup cell size is therefore generated by weighting the results of Equation 1 by the likelihood of each combination of *C*_*L*_ and *C*_*N*_ occurring in each specific sample, calculated using the frequency distribution of morphogroup *C*_*N*_ (from fossil coccospheres; Sections 2.3.2 and 2.3.3) and sample‐specific morphogroup *C*_*L*_ (loose coccolith measurements; Section 2.3.4).

Community size structure is a function of the cell size distribution of each species in the community and their relative cellular abundance in the assemblage. For each sample, we therefore normalized modeled morphogroup cell size frequency distribution by morphogroup relative cellular abundance, using mean *C*_*N*_ of the morphogroup to convert a five‐point moving average of relative coccolith abundance (calculated using the “smoothing spline” function in PAST4; Hammer et al., 2001) to relative cellular abundance. As a final step, the resultant abundance‐weighted histograms were stacked to produce the overall community cell size distribution of each sample.

#### 2.3.2 Fossil Coccosphere Data Set

We present a new data set of Eocene and Oligocene coccosphere geometry (Sheward, Gibbs, et al., 2024) quantified from over 2,000 individual, intact fossil coccospheres (e.g., Figure S2 in Supporting Information S1) from a range of globally distributed sites, including 375 Oligocene‐age coccospheres from Site U1553. Fossil coccospheres were observed using standard smear slides (Bown & Young, 1998) and examined under light microscopy at 1,000× magnification along continuous transects. Each intact fossil coccosphere observed was identified to species level if possible and imaged with a digital camera (image size 5,184 × 3,457 px with a resolution of 37.62 px μm^−1^ or 0.0266 μm px^−1^). The following morphometric data was collected from each coccosphere, following (Gibbs et al., 2013, 2018; Sheward et al., 2017): *C*_*N*_ was counted by finely adjusting focal depth, *C*_*L*_ was measured from an image focused on the distal surface of a representative coccolith on the upper coccosphere surface, and coccosphere diameter (i.e., including the coccolith layer, ∅) and cell diameter (internal coccosphere diameter, Θ) were measured from an image focused on the maximum coccosphere cross‐section (Table 1). Within‐genus counts of *C*_*N*_ were used to generate the frequency histogram of *C*_*N*_ for each genus used in the cell size model. Measurements were made using the freeware ImageJ (Schneider et al., 2012). Species within the Coccolithales and Isochrysidales are well‐represented in the data set but the data set does not contain coccosphere geometry data for *Discoaster*, *Sphenolithus*, or *Zygrhablithus* as their lith morphologies makes coccospheres of these taxa very susceptible to disarticulation (Bown et al., 2014).

#### 2.3.3 Estimating Cell Size in Non‐Placolith Taxa

As *Discoaster*, *Sphenolithus* and *Zygrhablithus* lack fossil coccosphere data, we have no direct observations for the frequency of *C*_*N*_ in these genera and cannot derive a relationship between Θ–*C*_*L*_–*C*_*N*_ as defined in Equation 1. The morphologies of *Discoaster*, *Sphenolithus* and *Zygrhablithus* coccoliths are relatively unique and without modern analogs that could constrain the relationship between cell size and other morphometric variables. However, observations of the coccosphere architecture of collapsed coccospheres with broadly similar morphological features and morphometric trait data sets for extant coccolithophores (Sheward et al., 2014, 2016, 2024) do highlight the following general characteristics: (a) across a wide diversity of species, within‐species *C*_*N*_ generally follows a Gaussian‐like distribution, and (b) the degree to which adjacent coccoliths abut (touching but not overlapping, thereby leaving some “gaps” in the coccosphere) or overlap each other to form the coccosphere influences the relationship between cell surface area and coccolith surface area.

Based on the assumption of a Gaussian‐like *C*_*N*_ frequency distribution, we simulated an artificial data set of *C*_*N*_ for each genus (Figure S5 in Supporting Information S1; further details in Text S3 in Supporting Information S1) generated from multiple random number generations defined by a *C*_*N*_ mean and standard deviation that was informed by the number of coccoliths observed in collapsed coccospheres with similar morphologies (Gibbs et al., 2018). Mean *C*_*N*_ was defined as 20 for *Discoaster* (simulated *C*_*N*_ range 10–31), 42.5 for *Sphenolithus* (simulated *C*_*N*_ range 27–60), and 22 for *Zygrhablithus* (simulated *C*_*N*_ range 9–37).

We then developed an alternative parameterization for the relationship between Θ, *C*_*L*_, and *C*_*N*_ from which Θ could be modeled (see also Text S3 in Supporting Information S1), based on the observation that coccolith surface area (a function of *C*_*L*_ and coccolith circularity) and cell surface area (a function of cell radius) are related by *C*_*N*_, following Equation 2:

(2) $$cell\;surface\;area\;\left({\mu m}^{2}\right)=C_{N}\times coccolith\;surface\;area\times {\;C}_{O}$$

where $C_{O}$ is the fraction of cell surface area that is either covered by overlapping adjacent coccoliths or associated with gaps between abutting coccoliths. The morphology of *Sphenolithus* and *Zygrhablithus* indicate that liths would have sat side‐by‐side on the cell surface with small gaps between them, comparable to extant holococcoliths and murolith forms. We assume that *Discoaster* nannoliths would completely cover the cell with a small degree of overlap, similar to extant species with circular‐shaped placolith or planolith coccoliths. For these genera, we can then alternately model cell size as a function of *C*_*L*_ and *C*_*N*_ combinations, following Equation 3:

(3) Θ(μm)=2CO×CN×CLP×CLPAR×π4π

where *C*_*N*_ is the number of coccoliths per cell, *C*_*LP*_ is the length (μm) of the coccolith proximal shield (i.e., “base” size for *Sphenolithus* spp. and *Z*. *bijugatus*), *AR* is the coccolith aspect ratio (ratio of mean *C*_*L*_ to mean coccolith width, *C*_*W*_), and *C*_*O*_ is the fraction of cell surface area increased by gaps between coccoliths or decreased by overlapping coccoliths (e.g., to account for overlapping coccoliths, a *C*_*O*_ of 0.8 would impose a 20% reduction in the cell surface area calculated from coccolith surface area multiplied by *C*_*N*_). As before, direct measurements of *C*_*L*_ frequency distribution and our simulated *C*_*N*_ frequency distributions for each genus were used to calculate the likelihood of each *C*_*L*_‐*C*_*N*_ combination occurring. These quantified likelihoods were summed to generate the frequency distribution of morphogroup cell size and normalized to morphogroup relative cellular abundance in each sample.

#### 2.3.4 Calcareous Nannoplankton Morphometrics

For each of the 18 samples used for reconstructing community traits, a data set of coccolith size (*C*_*L*_; *n* = 50 measurements) was collected for each morphogroup (Sheward, Herrle, et al., 2024) using light microscopy along a random transect of the microscope slide at 1,000× magnification. For taxa with placolith‐type coccolith morphologies, *C*_*L*_ was measured as the maximum length of the distal shield. As the distal shield rims of *Chiasmolithus* spp., *Cl*. *subdistichus*, and *Coccolithus* spp. coccoliths are formed of non‐birefringent V‐units, *C*_*L*_ was measured under plain light where the edge of the distal shield rim could be better observed. For *Discoaster*, nannolith size was measured as the diameter of a circle that encompassed the full extent of all rays and ray length was estimated as half of circle diameter. *Sphenolithus* (nannolith) and *Z*. *bijugatus* (holococcolithophore) base size and lith height were both measured, with base size used as the size input for the size model. *Discoaster*, *Sphenolithus* and Z. *bijugatus* only occurred sporadically in our samples and were generally rare. We therefore pooled size measurements from all samples into a single morphogroup size data set that was used for all 18 reconstructions (rather than a sample‐specific size data set, as for all other morphogroups).

#### 2.3.5 Cellular POC and PIC Traits: The Size‐Trait Model

We expanded the size model of Gibbs et al. (2018) to additionally model the distribution of cellular POC and PIC within each genus (here named the size‐trait model). Cell POC was estimated from the modeled cell radius of each *C*_*L*_‐*C*_*N*_ combination based on the cell biovolume to cell organic C relationship of Menden‐Deuer and Lessard (2000) for Prymnesiophytes, following Equation 4:

(4) $$cellular\;POC\;\left(pg\;C\right)={0.228\left(cell\;volume\right)}^{0.899}$$

We estimated cellular PIC for each *C*_*L*_‐*C*_*N*_ combination based on the well‐established morphometric‐based approach of Young and Ziveri (2000), following Equation 5:

(5) $$cellular\;PIC\;\left(pg\;C\right)=coccolith\;PIC\times C_{N}$$

where *C*_*N*_ is number of coccoliths per cell and coccolith PIC is calculated as a function of coccolith morphology and cross‐sectional thickness, following Equation 6:

(6) $$coccolith\;PIC\;\left(pg\;C\right)=\left({C_{L}}^{3}\times K_{s}\times 2.7\right)\times 0.12$$

where *C*_*L*_ is coccolith size (μm), *Ks* is a morphogroup‐specific shape factor related to coccolith cross‐sectional shape, and 2.7 is the mass of calcite (pg CaCO_3_ μm^3^). We convert the result from pg CaCO_3_ to pg C, assuming that the mass of carbon is 12% of the mass of CaCO_3_. We used published, genus‐specific *Ks* values where available (Agnini et al., 2016; Preiss‐Daimler et al., 2012; Young & Ziveri, 2000) or otherwise adapted the published *Ks* values of species with similar morphologies (Gibbs et al., 2018). The *Ks* values used for each morphogroup are listed in Table 2 and described further in Text S4.

**Table 2:** Morphogroup Biogeochemical Traits at Site U1553 During the Latest Eocene and Oligocene

	Model parameters			Cellular^a^				100‐Cell population^b^	
Morphogroup	Cell size^c^	*Ks* ^d^		Cell size (μm)	POC (pg C cell^−1^)	PIC (pg C cell^−1^)	PIC:POC (mol:mol)	Total POC (pg C)	Total PIC (pg C)
Placolith‐bearing morphogroups:									
*Chiasmolithus*	*α* = 0.478	0.06	Mean	15.7	232	337	1.46	23,413	32,752
	*β* = 0.151		Range	13.2–17.4	146–303	183–475	1.22–1.63		
			5th to 95th	11.5–21.5	105–465	105–675	1.05–1.85		
*Coccolithus*	*α* = 0.623	0.06	Mean	13.22	149	177	1.19	15,243	18,082
	*β* = −0.122		Range	10.4–14.7	80–204	91–231	1.04–1.21		
			5th to 95th	8.5–18.5	45–345	45–375	1.05–1.35		
*Clausicoccus*	*α* = 0.513	0.05	Mean	14.03	171	70	0.43	16,800	7,039
	*β* = −0.025		Range	‐	‐	‐	‐		
			5th to 95th	10.5–17.5	75–285	30–105	0.35–0.55		
*Cyclicargolithus*	*α* = 0.441	0.08	Mean	8.54	48	125	2.65	4,882	12,551
	*β* = 0.275		Range	6.4–10.4	20–78	55–198	2.54–2.74		
			5th to 95th	5.5–12.5	15–105	45–285	2.35–2.95		
*R*. *bisecta g*roup	*α* = 0.574	0.07	Mean	10.14	76	164	2.00	7,577	16,447
	*β* = 0.052		Range	8.1–11.8	38–107	71–247	1.69–2.25		
			5th to 95th	6.5–15.0	15–195	45–465	1.45–2.65		
*R*. *lockeri* group	*α* = 0.574	0.05	Mean	8.44	42	53	1.25	4,190	5,255
	*β* = 0.052		Range	7.8–8.9	34–50	39–62	1.16–1.36		
			5th to 95th	6.5–10.5	15–75	15–105	1.05–1.55		
*R*. *umbilicus g*roup	*α* = 0.574	0.045	Mean	7.33	40	55	1.01	4,063	5,622
	*β* = 0.052		Range	5.1–12.1	16–137	16–234	0.79–1.46		
			5th to 95th	4.5–14.5	15–165	15–285	0.65–1.75		
Nannolith‐ and holococcolith‐bearing morphogroups:									
*Sphenolithus* (nannolith)	*C*_*O*_ = 1.18	0.05 (base)	Mean	14.39	119	38	0.32	18,900	5,352
			Range	‐	‐	‐	‐		
			5th to 95th	9.5–20.5	45–255	15–105	0.25–0.35		
*Discoaster* (nannolith)	*C*_*O*_ = 0.80	0.22 (ray length)	Mean	22.42	437	263	0.45	65,334	29,627
			Range	‐	‐	‐	‐		
			5th to 95th	14.5–33.5	165–825	45–765	0.35–0.55		
*Z*. *bijugatus* (holococcolith)	*C*_*O*_ = 1.13	0.40 (height)	Mean	18.91	321	604	3.24	40,379	127,024
			Range	‐	‐	‐	‐		
			5th to 95th	11.5–26.5	75–675	195–945	2.85–3.85		

^a^ Mean and 5th–95th percentile values are derived from data sets of coccosphere geometry and all morphogroup *C*_*L*_ integrated into the size‐trait model. Range is the range of mean morphogroup trait values across all 18 reconstructions for morphogroups with more than three sample‐specific *C*_*L*_ data sets.

^b^ Total population POC and PIC are reported for a hypothetical 100‐cell population of each morphogroup.

^c^ The calculation of cell size from coccosphere geometry takes the form log_10_(*C*_*L*_) = *α*[log_10_ (cell surface area/*C*_*N*_)] + β (Equation 1) or is based on the relationship between coccolith surface area and cell surface area proportional to assumed degree of coccolith overlap, *C*_*O*_, for nannolith‐ and holococcolith‐bearing morphogroups (Text S3 in Supporting Information S1).

^d^ the calculation of cellular PIC uses a shape factor, *Ks* (Text S4 in Supporting Information S1).

Following a similar process as for cell size, the cellular POC and PIC values of each *C*_*L*_‐*C*_*N*_ combination were weighted by the likelihood of occurrence based on the frequency distribution of *C*_*L*_ and *C*_*N*_ in the sample. Weighted values were summed within each cell size class to produce the equivalent distribution of POC or PIC across size classes for each morphogroup. Our modeled cellular PIC results show good agreement with the range of morphometric‐based estimates of cellular PIC for individual fossil coccospheres in the same cell size range (Figure S7 in Supporting Information S1).

Morphogroup values for “total community” POC and PIC (Table 1) were then normalized to morphogroup relative cellular abundance (as for community cell size distribution). Estimates of true total community POC and PIC (e.g., pg C mL^−1^) scale with both species carbon content and absolute abundance (cells mL^−1^), which is strongly regulated by nutrient availability and productivity (e.g., Chavez et al., 2011). However, we do not attempt to scale our modeled community‐level POC or PIC results to estimated fluctuations in carrying capacity through time as geochemical proxies for the nutrient regime/paleoproducivity at Site U1553 are not yet available. Instead, our analysis assumes (unrealistically) that the carrying capacity at Site U1553 remained constant through the study interval at a community standing stock of 100 cells for all samples (i.e., total community POC here represents pg C per 100 cells), guided by the abundance of coccolithophores typical for present‐day temperate shelf seas (Mayers et al., 2017). As such, we focus our discussion on the model results that are independent of changes in carrying capacity through time: the cellular PIC:POC of each morphogroup, shifts in POC and PIC distribution between size classes, and trends in overall community PIC:POC.

## 3 Results

### 3.1 Latest Eocene and Oligocene Assemblage Composition at Site U1553

**Figure 1:**
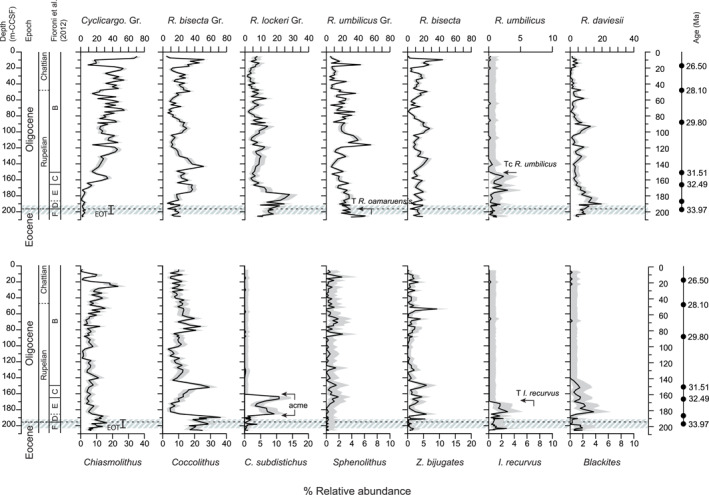
Relative abundance (%) of calcareous nannoplankton at Site U1553. Gray envelopes are 95% confidence intervals for each species, genus or morphogroup. Gr. = group. The high latitude zonation scheme of Fioroni et al. (2012) is shown: zone B = *Chiasmolithus altus* Zone, zone C = *Reticulofenestra daviesii* Zone, zone D = *Blackites spinosus*, zone E = *Reticulofenestra samodurovi* Zone, zone F = *Reticulofenestra oamaruensis* Zone. The T *R*. *oamaruensis* at 33.97 Ma marks the Eocene‐Oligocene Boundary (EOB) in high latitudes (Fioroni et al., 2012; dashed line). The timing of the Eocene‐Oligocene Transition (EOT, 34.44–33.65 Ma; Hutchinson et al., 2021) is indicated based on our age model (Table S1 in Supporting Information S1). Age tie‐points for the bioevents (GTS 2012) are shown on the right of the figure.

Based on the age‐depth tie‐points of identified bioevents (Text S1; Table S1 in Supporting Information S1), our record spans the latest Eocene to mid‐late Oligocene, 34.7 to 26.5 Ma. The calcareous nannoplankton community at Site U1553 (Figure 1) was dominated throughout by species in the genera *Cyclicargolithus*, *Reticulofenestra*, *Coccolithus*, and *Chiasmolithus* that together comprised 70%–95% of the assemblage. One of the most notable trends in assemblage composition is the progressive increase in *Cyclicargolithus* relative abundance through the record, from <10% during the latest Eocene and earliest Oligocene (ca. 34.7–31.2 Ma), to between 20% and 70% of the assemblage for the remainder of the Oligocene. This trend is also highlighted in the NMDS analysis (N sample depths = 90, N morphogroups = 10, N assemblages = 5, N dimensions = 3, stress value = 0.13, trymax = 5,000) and subsequent ANOSIM (ANOSIM‐R = 0.62, *p* < 0.001) (Figure S8 in Supporting Information S1), which confirms that the composition of mid‐late Oligocene communities (Assemblages 3–5) were distinctly different from those of the latest Eocene and earliest Oligocene (Assemblages 1–2). Also contributing to dissimilarities in community composition between the latest Eocene/EOT (Assemblage 1) and early Oligocene (Assemblage 2) are an abundance acme in *Cl*. *subdistichus* between ca. 183 and 164 m‐CCSF (33.26–32.36 Ma), a peak in *R*. *daviesii* abundance (up to 20% at 190 m‐CCSF), and a relatively elevated abundance of *Chiasmolithus* (10%–16% abundance at 188 m‐CCSF). *Coccolithus* and *Chiasmolithus* were persistent components of the assemblage throughout the record (3%–36% and 4%–12%, respectively) and *Coccolithus* shows two sharp decreases in abundance, the first around the Eocene‐Oligocene Boundary (EOB) and the second in the early Oligocene. The remaining taxa (including *Sphenolithus*, *Discoaster*, *Z*. *bijugatus*, *Ismolithus recurvus*) account for <10% of the assemblage, averaging 3%.

### 3.2 Cell Size and Biogeochemical Traits of Oligocene Taxa

**Figure 2:**
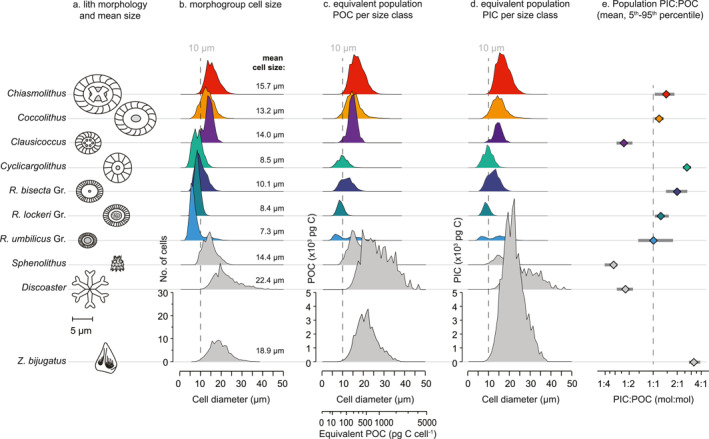
Cellular biogeochemical traits of late Eocene‐Oligocene calcareous nannoplankton morphogroups used in this study. (a) mean coccolith size and illustrative morphology, (b) modeled cell size (μm), (c) distribution of cellular particulate organic carbon (POC, pg C cell^−1^) content across size classes, (d) distribution of cellular particulate inorganic carbon content (PIC, pg C cell^−1^) across size classes, (e) morphogroup PIC:POC (mol:mol). The frequency distribution of cell size (b), POC (c) and PIC (d) are visualized for a hypothetical population of 100 cells mL^−1^ to emphasize differences in morphogroup POC and/or PIC content for the same number of cells (see also Table 2).

Across the 10 morphogroups investigated, modeled cell size (Figure 2, Table 2) ranged from a minimum of 2.3 μm (*R*. *minuta* in *R*. *umbilicus* group) to a maximum of 46.2 μm (*Discoaster*) for a total *C*_*L*_ range of 2.27–18.74 μm across all samples and morphogroups. Overall, 50% of the modeled cell sizes were between 8.5 and 15.5 μm and 90% between 5.5 and 22.5 μm (weighted by frequency of liths in each *C*_*L*_ size fraction but not by morphogroup relative abundance). Noelaerhabdaceae morphogroups (*Cyclicargolithus* and *Reticulofenestra*) were generally smaller (mean cell size 7–10 μm) than Coccolithaceae morphogroups (*Chiasmolithus*, *Coccolithus*, and *Clausicoccus*; mean cell size 13–16 μm). In *Chiasmolithus*, *Coccolithus* and *R*. *umbilicus* group, maximum cell size is more than double the 95th percentile of the cell size (Size_95_), reflecting the presence of species with very large coccoliths (>14 μm) during this interval, for example, *R*. *umbilicus*, *R*. *hillae*, and *Coccolithus eopelagicus*. Overall, the largest mean cell sizes (14–22 μm) were modeled for the nannolith groups *Sphenolithus*, *Discoaster* and the holococcolithophore *Z*. *bijugatus*.

Cellular POC content varied between morphogroups, reflecting differences in modeled cell volume. Mean cellular POC was lowest in the Noelaerhabdaceae (40–76 pg C cell^−1^) and largest in *Sphenolithus*, *Discoaster* and *Z*. *bijugatus* (119–437 pg C cell^−1^) (Figure 2; Table 2). Cellular PIC content was estimated as a function of *C*_*L*_ and *C*_*N*_ (Methods), therefore larger cells were associated with larger cellular PIC in both intact fossil coccospheres and the model results (Figure S7 in Supporting Information S1). Cellular PIC content was lowest in *Sphenolithus* (mean = 38 pg C cell^−1^, range = 9–127 pg C cell^−1^) and greatest in *Z*. *bijugatus* (mean = 604 pg C cell^−1^, range = 82–4,683 pg C cell^−1^). Mean cellular PIC:POC varied substantially between morphogroups (Figure 2e; Table 2), showing an order of magnitude difference between *Sphenolithus* (mean PIC:POC of 0.32) and *Z*. *bijugatus* (mean PIC:POC of 3.2). The majority of cells in *Chiasmolithus* (57%), *Coccolithus* (100%), *R*. *lockeri* group (93%) and *R*. *umbilicus* group (91%) had a PIC:POC between 0.5 and 1.5 (1:2 to 2:1; Figure 2). In contrast, cells of *Clausicoccus*, *Sphenolithus* and *Discoaster* morphogroups had a persistently low PIC:POC of ca. 0.25 to 0.55 whereas *Cyclicargolithus*, *R*. *bisecta*, and *Z*. *bijugatus* coccospheres were notably “heavily calcified” relative to their cell size (*Cyclicargolithus* PIC:PIC range = 1.95–3.28; *Z*. *bijugatus* PIC:POC range = 2.49–4.7). Morphogroup cell size and cellular PIC:POC did not fluctuate significantly through time (Figure S9 in Supporting Information S1) as *C*_*L*_ was relatively constant for most morphogroups through the Oligocene and cellular POC and PIC tended to vary in concert, as both traits are modeled as functions of the same *C*_*L*_ and *C*_*N*_ data sets. The notable exception to this is the significant cell size decrease in *R*. *umbilicus* Group in the early Oligocene after the extinction of the very large species *R*. *umbilicus*, which drives an order of magnitude decrease in cellular POC and PIC, and a mean PIC:POC decrease from a peak of 1.46 to 0.8–1.0 for the remainder of the Oligocene.

### 3.3 Community Size Structure and PIC:POC

**Figure 3:**
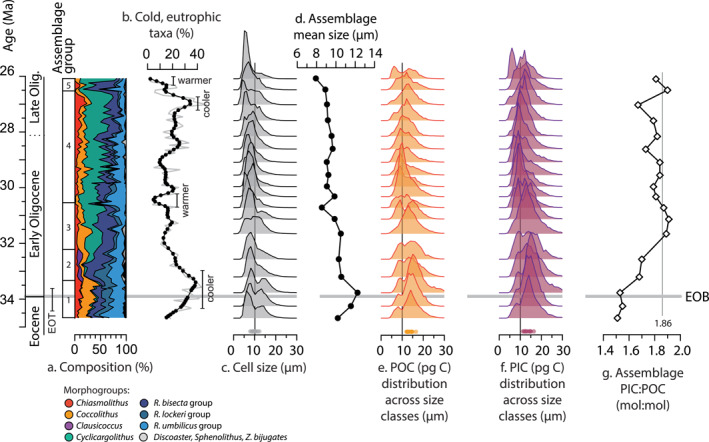
Calcareous nannoplankton biogeochemical traits at Site U1553 through the late Eocene and Oligocene for a fixed‐abundance community (100 cells). (a) Community composition and labeled assemblage groups 1–5. (b) Percentage of cold, meso‐eutrophic taxa (*Chiasmolithus*, *R*. *daviesii* and *Cl*. *subdistichus*; e.g., Viganò et al., 2023, 2024), 5‐point smoothing applied to relative abundance (black), with inferred cooler (>30% cold taxa) and warmer (<10% cold taxa) intervals indicated. (c) Community size structure with reference 10 μm cell size (black line). (d) Mean community cell size. (e) Distribution of POC across size classes. (f) Distribution of PIC across size classes. (g) Community PIC:POC. The EOB (33.97 Ma, T *R*. *oamaruensis*) is shown by horizontal gray line. The approximate duration of the Eocene‐Oligocene transition (EOT) is indicated based on our age‐model (GTS 2012).

The cell size distribution of the latest Eocene and Oligocene community at Site U1553 (Figure 3) is consistently skewed toward small‐intermediate size classes (mean community size 8–12.5 μm, 80% of cells between 3–4 μm and 11–16 μm). This is unsurprising given the high abundance of small *Reticulofenestra* and *Cyclicargolithus* throughout the record. Although large and very large cells >15 μm are a minor component of the community (averaging ca. 5%–10%), cells >15 μm comprise ca. 25% of the community during the latest Eocene and earliest Oligocene due to an increased presence of large and very large *Chiasmolithus*, *Coccolithus* and *Reticulofenestra* (Figure S9 in Supporting Information S1). Mean community size was subsequently largest (12.4 μm) in the earliest Oligocene, coincident with the timing of the EOT based on our age model. The extinction of *R*. *umbilicus* and declining abundance of another large species, *R*. *hillae*, combined with the rising abundance of small *Cyclicargolithus* from the earliest Oligocene onwards led to a gradual decrease in mean community size through the early‐mid and mid‐late Oligocene, with a minimum mean community size of 7.9 μm at the top of our record (Figure 3d).

**Figure 4:**
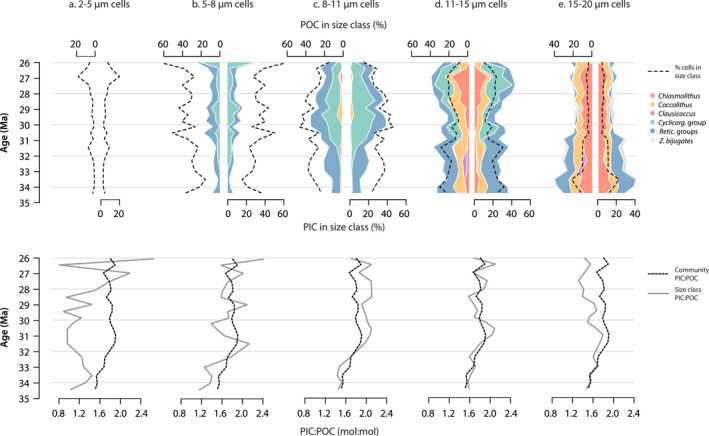
Community PIC:POC within size classes (a) 2–5 μm, (b) 5–8 μm, (c) 8–11 μm, (d) 11–15 μm, and (e) 15–20 μm. Top panel: stack plots show the percentage of total community POC (left) and PIC (right) within each size fraction separated into morphogroup by color. The relative cellular abundance in each size class through time is shown by the dashed black line. Bottom panel: PIC:POC of each size fraction (gray line) and overall community PIC:POC (black dashed line).

Community PIC:POC always exceeded 1.5 (i.e., minimum 1.5 times inorganic carbon than organic carbon), as the most abundant morphogroups at Site U1553 were generally heavily calcified (Figure 2, Table 2). Community PIC:POC shows an opposite trend to mean cell size, increasing through the record from a minimum of ca. 1.5 in the latest Eocene‐earliest Oligocene to a maximum of 1.9 in the Early Oligocene, after which PIC:POC fluctuates at 1.8–1.9 for the remainder of the record (Figure 3g). Increasing community PIC:POC through time reflects the high mean PIC:POC of *Cyclicargolithus* (2.5–2.7) and *R*. *bisecta* group (1.7–2.3) that together represent 43%–72% of the calcareous nannoplankton community from the early Oligocene onwards (Figure 1) combined with a ca. 30% decrease in the PIC:POC of *R*. *umbilicus* group in the early Oligocene (Figure S9 in Supporting Information S1). The three rare morphogroups in the model—*Sphenolithus* (<3% abundance), *Discoaster* (<1% abundance), and *Z*. *bijugatus* (typically <5% abundance)—rarely contributed more than 5% to community POC and PIC (averaging ca. 1% of total POC and PIC for 100 cells) and therefore had negligible impact on community PIC:POC. Overall community PIC:POC most closely tracks PIC:POC trends within the 8–11 μm and 11–15 μm size classes (Figure 4) that together contain on average 50%–70% of cells in the community. However, up to 60% of the total POC and PIC in the community is partitioned into the 11–20 μm size classes that represent less than 25% of the community on average (Figure 4). The greater POC and PIC content of larger cells/taxa (e.g., *Coccolithus*, *Chiasmolithus*, and large Noelaerhabdaceae species) means that they contribute a disproportionate fraction of total POC and PIC relative to their abundance in the community whilst smaller taxa contribute proportionally less to total community POC and PIC than might be expected from their higher relative abundance.

## 4 Discussion

### 4.1 Community Composition Under Latest Eocene and Oligocene Climate

The long‐term transition from early Paleogene greenhouse conditions to Neogene icehouse conditions is one of the most significant features of Cenozoic climate and was associated with major changes in marine and terrestrial environmental conditions. Decreasing atmospheric CO_2_ concentrations and evolving continental configurations are hypothesized to have driven long‐term global cooling beginning in the early mid Eocene (e.g., Anagnostou et al., 2016; Bijl et al., 2013; Inglis et al., 2015; Pagani et al., 2005; Sauermilch et al., 2021; Westerhold et al., 2020; Zachos et al., 2001), cumulating in late Eocene climate instability, shifts in latitudinal temperature gradients, and the initiation of major sustained continental‐scale ice sheets in Antarctica beginning in the EOT and early Oligocene glacial maximum (EOGM) (e.g., Hutchinson et al., 2021). In the southern high latitudes, proxies indicate surface water cooling of up to 1–8°C between the late Eocene and early Oligocene alongside changes in water column stratification and nutrient availability (Hutchinson et al., 2021 and references therein). All major microfossil groups show biotic responses to the EOT or shortly thereafter, including diversity declines, extinctions, temperature‐related shifts in abundance and/or biogeography, transient species occurrences, and/or morphological changes (e.g., Cotton & Pearson, 2011; Coxall & Pearson, 2007; Dunkley Jones et al., 2008; Funakawa et al., 2006; Pascher et al., 2015; Pearson et al., 2008; Swain et al., 2024; Wade & Pearson, 2008). Calcareous nannoplankton assemblage records spanning the late Eocene‐early Oligocene show significant decreases in nannoplankton diversity (Bown et al., 2004; Lowery et al., 2020), dramatic shifts in Southern Ocean and higher latitude assemblage composition (Persico & Villa, 2004; Viganò et al., 2024; Villa et al., 2008) and marked declines in the abundance of oligotrophic taxa in lower latitudes (Blaj et al., 2009; Bordiga et al., 2015; Dunkley Jones et al., 2008; Jones et al., 2019). As most previous work has concentrated on the EOT specifically, comparatively little is known about the composition of Oligocene assemblages more generally. Our record of assemblage composition at Site U1553 does not focus on the detailed impact of the EOT on calcareous nannoplankton but rather captures the broad features of the assemblage response to climatic disruption across the latest Eocene and earliest Oligocene within the context of long‐term trends in assemblage composition driven by millennial‐scale climate evolution through the greenhouse‐icehouse transition.

The latest Eocene and Oligocene calcareous nannoplankton community at Site U1553 was dominated by *Cyclicargolithus* and *Reticulofenestra* with low to moderate abundances of *Chiasmolithus* and *Coccolithus* (ca. 20% community; Figure 1). The inferred paleoecologies of these taxa indicate a predominantly temperate assemblage at Site U1553 and rare abundances of warm‐water species (*Discoaster*, *Sphenolithus*) suggest a limited influence of warm, subtropical‐tropical waters. The moderate abundance (rather than dominance) of cold‐water taxa (*Chiasmolithus*, *R*. *daviesii*) indicate that Site U1553 was not strongly influenced by cold waters originating in the subantarctic and Antarctic regions, in comparison to records from Southern Ocean sites Kerguelen Plateau and Maud Rise where these taxa are more abundant (Persico & Villa, 2004; Villa et al., 2008, 2014). Overall, our assemblage record suggests that Site U1553 was mostly situated north of the proto‐SAF during our record, in agreement with paleoceanographic reconstructions of the region based on a range of biogenic and geochemical proxies (Hoem et al., 2021; Kamp et al., 1990; Nelson & Cooke, 2001; Pascher et al., 2015; Sarkar et al., 2019; Scher et al., 2015). Assemblage composition at Site U1553 is similar to the mid‐latitude Tasman Sea (IODP Site U1509), which was also dominated by *Reticulofenestra*, *Cyclicargolithus* and *Coccolithus* and was likely to have been situated north of the proto‐STF during this time (Viganò et al., 2024).

**Figure 5:**
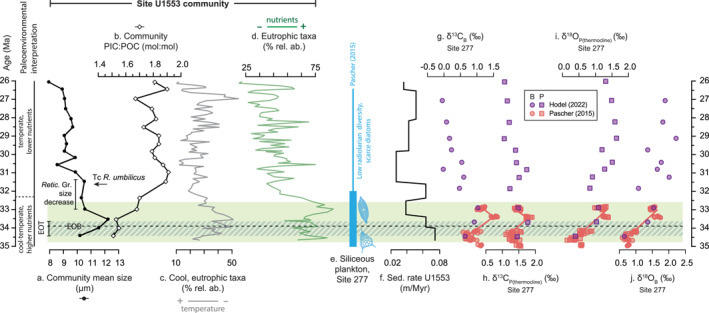
Calcareous nannoplankton community traits and composition within the paleoenvironmental context, Site U1553 (this study) and Site 277 (Campbell Plateau). (a) Mean community cell size. (b) Community PIC:POC. (c) Percentage of cool, eutrophic taxa (*Chiasmolithus*, *R*. *bisecta*, *Cl*. *subdistichus*). (d) Percentage of eutrophic taxa (*Chiasmolithus*, *Cl*. *subdistichus*, *I*. *recurvus*, *R*. *lockeri* Gr., *R*. *umbilicus* Gr.). (e) Interval of high radiolarian and diatom abundance and abundant high latitude radiolarian taxa at Site 277, ca. 35–32 Ma, followed by an interval of declining radiolarian diversity and scarce diatoms (Pascher et al., 2015). (f) Sedimentation rate at Site U1553, estimated based on our age model (Table S1 in Supporting Information S1). (g–j) Benthic (*Cibicidoides* spp.) and planktonic (*Subbotina* spp., a thermocline‐dwelling genus) foraminifera oxygen and carbon isotope data from DSDP Site 277 from Pascher et al. (2015) and Hodel et al. (2022). (g) Benthic δ^13^C values (δ^13^C_B_). (h) Planktonic δ^13^C values (δ^13^C_P_). (i) Planktonic (δ^18^O_P_) oxygen isotopes. (j) Benthic (δ^18^O_B_) oxygen isotopes. The postulated interval of cooler, higher nutrient conditions at Site U1553 is represented by the green shaded area. Data are plotted using the age model for U1553 or the depths of bioevents reported in Hodel et al. (2022) for DSDP Site 277 data from Hodel et al. (2022) and Pascher et al. (2015), following Gradstein et al. (2012).

Shifts in community composition at Site U1553 across the latest Eocene and earliest Oligocene indicate an interval of cooler sea surface temperatures (SSTs) and enhanced surface water nutrients (Figures 3b and 5) that are generally consistent with regional and high‐latitude observations of declining abundances of warm‐oligotrophic taxa and increased abundances of cold‐eutrophic taxa across the EOT and EOGM (e.g., Viganò et al., 2024; Villa et al., 2014). We record an acme of *Cl*. *subdistichus* (potentially a eutrophic taxon; Viganò et al., 2023) between 33.26 and 32.36 Ma, elevated abundances of cold‐water taxa *Chiasmolithus* spp.and *R*. *daviesii* (also recognized at or shortly after the EOB at high southern latitudes; Fioroni et al., 2012; Persico & Villa, 2004; Villa et al., 2008), an earliest Oligocene presence of *Blackites* spp. (inferred cool and potentially mesotrophic preference, e.g., Kochhann et al., 2021), and an abundance decrease in the warm‐water taxa *Coccolithus* (Figure 1). In future, high resolution assemblage and geochemical records would allow the timing of these events to be correlated with shifts in paleoenvironmental conditions across the EOT and EOGM.

Following the earliest Oligocene, the most prominent feature of the assemblage composition at Site U1553 through time is the gradual increase in *Cyclicargolithus* abundance up‐core through the record (Figure 1). The paleoecology of *Cyclicargolithus* evolved from a warm‐temperate affinity during the Eocene (Newsam et al., 2016) to an increasingly widespread and cosmopolitan distribution and meso‐eutrophic preference through the Oligocene (Dunkley Jones et al., 2008; Kochhann et al., 2021; Toffanin et al., 2011; Wei & Wise, 1990). This *Cyclicargolithus* abundance trend may therefore indicate a transition away from cool conditions across the EOT and EOGM towards increasingly temperate, meso‐eutrophic conditions with a minor component of cool‐cold water taxa through the Oligocene (Figures 3 and 5).

Superimposed on the long‐term trend of increasing *Cyclicargolithus* abundance are additional indicators of oscillating intervals of warming and cooling temperatures (Figure 3b). The timing of these features (based on our age model) are generally consistent with the timing of Oligocene paleotemperature records. An interval of warmer conditions is indicated in the early Oligocene between ca. 30.3–32.0 Ma by a peak in warm‐water *Coccolithus* (ca. 31.6 Ma), the extinction of very large *R*. *umbilicus*, and an interval of low abundances of cool‐eutrophic taxa. This is in agreement with a compilation of high southern latitude records, which show that cool earliest Oligocene SSTs somewhat rebound to warmer temperatures between ca. 31.5 and 30.5 Ma (O’Brien et al., 2020). Higher abundances of cold‐water *Chiasmolithus* (10%–35%) between ca. 28.3–26.6 Ma (Figure 1) suggest an interval of cooler conditions during the mid‐Oligocene that is broadly consistent with the timing of the Mid‐Oligocene Glacial Interval first defined in the mid‐Atlantic Ocean (ca. 28.0–26.3 Ma; Liebrand et al., 2017). A notable decrease in the abundance of cool, eutrophic taxa is also evident in the uppermost portion of our record (Figure 3b) and may relate to the onset of Late Oligocene warming beginning ca. 26.5 Ma (e.g., Liebrand et al., 2017; O’Brien et al., 2020; Villa et al., 2014; Villa & Persico, 2006).

The diversity of the assemblage at Site U1553 is low (12–29 species, highest number of species in latest Eocene and earliest Oligocene then declining) but consistent with low late Eocene and early Oligocene diversity reported at other regional and higher latitude sites (e.g., Madile & Monechi, 1991; Viganò et al., 2024) and the early Oligocene minimum in calcareous nannoplankton diversity and evolution rates (Bown et al., 2004). Although preservation at Site U1553 is overall very good to moderate through our record (including intact coccosphere preservation and consistent presence of small placoliths), the presence of lower assemblage diversity, evidence of calcite overgrowth, fragmentation, loss of *Chiasmolithus* central area crosses, and clear dominance of robustly calcified taxa in the assemblage warrant consideration of the influence of diagenetic biases on our record. Deep‐sea sediments tend to underrepresent dissolution‐susceptible species with very small coccoliths (<3 μm), very lightly calcified coccoliths, or holococcoliths (Young et al., 2005) resulting in poor fossil records for species with these lith characteristics (Bown et al., 2008). Diversity loss might especially be an issue during the late Eocene, where exceptionally well‐preserved low latitude continental shelf assemblages are two‐to three‐times more diverse than their contemporaries at other carbonate‐rich open‐ocean sites (Bown et al., 2008). However, we note that Paleogene species diversity is less strongly skewed towards smaller coccolith sizes than present‐day diversity (thus limiting diversity loss of very small taxa; Bown et al., 2008) and the majority of species diversity in present‐day coccolithophore assemblages, even at low latitudes, is within rare taxa (Poulton et al., 2017). Additionally, dissolution‐susceptible taxa tend to be either very small or very lightly calcified (or both) with correspondingly low cellular POC and/or PIC (e.g., Sheward et al., 2024 for extant taxa). They are therefore unlikely to be major contributors to community calcite or biomass production unless present in extremely high abundances relative to other taxa (Daniels et al., 2016), although the contribution of rare extant taxa with low cellular POC and/or PIC to community biomass and calcite has not been quantified. On balance, we therefore assume that any loss of diversity due to selective preservation would have a relatively small impact on our record of assemblage composition and not significantly change our interpretation of the size and biogeochemical traits of the community.

### 4.2 Response of Community Composition to Climate Affects Community Size Structure and Biogeochemical Traits

Temporal variability in community size structure and community PIC:POC at Site U1553 is driven by changes in community composition that impact the mix of biogeochemical traits in the community: a shift in *Reticulofenestra* size diversity (Figure S9 in Supporting Information S1) and particularly the loss of large *Reticulofenestra* species (*R*. *umbilicus*, *R*. *hillae*) in the early Oligocene (also noted by Henderiks & Pagani, 2008), the increasing abundance of highly calcified, small‐medium‐sized cells of *Cyclicargolithus* beginning in the early Oligocene, and varying abundances of larger *Coccolithus* and *Chiasmolithus*. These trends in community composition result in larger cell sizes and size diversity across the EOT and earliest Oligocene followed by a notable decrease in mean and largest cell sizes coupled with a trend of increasing community PIC:POC through the Oligocene (Figures 3 and 5).

Trends in assemblage composition at Site U1553 indicate that the interval of largest mean community size and size diversity in the latest Eocene‐earliest Oligocene may have been associated with an interval of cooler conditions and elevated nutrients (Figure 5). This interpretation is supported by evidence for elevated sedimentation rates ca. 33.4 to 34.7 Ma (Figure 5; Röhl et al., 2022) in the latest Eocene and earliest Oligocene (potentially indicating enhanced export productivity) and the presence of high latitude silicoflagellate species *Stephanoca speculum* at the EOB, interpreted as indicating a shelf upwelling setting (McCartney et al., 2023). At DSDP Site 277 (previously drilled at the same locality as Site U1553), Pascher et al. (2015) report greater diatom abundance and radiolarian diversity in the uppermost Eocene and lowermost Oligocene (ca. 35.3 to 32.4 Ma) attributed to a shift in the position of the proto‐Subantarctic Front (SAF) bringing cooler, more nutrient rich water masses across Campbell Plateau (Pascher et al., 2015). Low resolution oxygen isotope records from Site 277 (Hodel et al., 2022; Pascher et al., 2015) also show a sharp increase in the δ^18^O of both planktonic and benthic foraminifera ca. 34.5 and 33 Ma (Figure 5), indicating an overall cooling of bottom water and thermocline temperatures on Campbell Plateau across the EOT and/or continental ice growth (e.g., Lear et al., 2008). The approximately 1‰ positive benthic δ^13^C excursion at Site 277 (Figure 5; Hodel et al., 2022; Pascher et al., 2015) also suggests a change in the carbon cycle across this interval.

There is general consensus that surface nutrient availability increased and temperatures cooled across the southern high latitudes during the Late Eocene and into the early Oligocene (Bordiga et al., 2015; Diester‐Haass et al., 1996; Diester‐Haass & Zahn, 2001; Dunkley Jones et al., 2008; Fioroni et al., 2012; Jones et al., 2019; Persico & Villa, 2004; Villa et al., 2008, 2014, 2021), leading to enhanced high latitude primary production (Anderson & Delaney, 2005; Diester‐Haass & Zahn, 1996; Latimer & Filippelli, 2002; Rodrigues de Faria et al., 2024; Salamy & Zachos, 1999). In the Australian‐New Zealand subantarctic region, nutrient supply is likely to have responded to Tasman Gateway opening and the progressive development of a weak, proto‐Antarctic circumpolar current during the late Eocene and Oligocene (Kennett et al., 1975; Sarkar et al., 2019; Sauermilch et al., 2021; Scher et al., 2015). This is proposed to have altered the strength and location of the proto‐Subtropical Front (STF) and prevailing ocean currents in the region (Hodel et al., 2022; Hoem et al., 2021, 2022; Pascher et al., 2015), thus enhancing the upwelling of more nutrient‐enriched water masses in the middle and high latitudes of the South Pacific in the Early Oligocene (Viganò et al., 2024). A comprehensive assessment of paleoproductivity at U1553 is currently not possible due to the limited availability of paleoproductivity and geochemical data sets at the site. However, based on trends in our assemblage record within the context of regional paleoceanography (Figure 5), we postulate that the interval of high size diversity and increased mean community cell size recorded at Site U1553 supports existing evidence for increased nutrient availability across the EOT in this region.

An interpretation of elevated nutrient availability supporting larger cell sizes is generally consistent with the size‐scaling of many aspects of phytoplankton physiology and core metabolic processes (e.g., Beardall et al., 2009; Finkel et al., 2010). Larger phytoplankton cells have greater cellular C, N, and P content and therefore a higher nutrient requirement (an allometric relationship that we have, in part, explicitly parameterized in the model as a log‐log linear biovolume to cell carbon relationship; e.g., Menden‐Deuer & Lessard, 2000; Villiot et al., 2021). Nutrient uptake and growth rates also show size‐dependency (e.g., Edwards et al., 2012; Marañón et al., 2013). This potentially disadvantages larger microphytoplankton under low nutrient conditions because their volume‐specific uptake rate is inversely proportional to cell radius (Chisholm, 1992; Menden‐Deuer & Kiørboe, 2016) and they are less efficient at converting nutrients into biomass (Marañón et al., 2013). Whilst these nutrient‐physiology “size scaling laws” present a greatly simplified perspective, they are a useful and widely used framework to understand nutrient availability as one “bottom up” regulator of spatial and temporal patterns in plankton community size structure. For example, modern stratified, oligotrophic settings tend to be dominated by picophytoplankton <2 μm whilst eutrophic settings tend to support greater size diversity and larger phytoplankton, for example, diatoms >20 μm (e.g., Agawin et al., 2000; Dutkiewicz et al., 2020; Marañón et al., 2001).

At Site U1553, one explanation for the greater abundance of larger cells in the community in the earliest Oligocene is that increased nutrient availability relaxed selective pressures on larger cells for nutrients. Likewise, a relative reduction in nutrient supply following the EOT may have contributed to the extinction of several very large species (e.g., *R*. *umbilicus*) in the early mid Oligocene. A shift towards lower nutrient availability following the EOT is further suggested by the lower diversity of radiolarian assemblages and scarce diatoms recorded at Site U1553 (ca. 130–180 mbsf; Röhl et al., 2022) and Site 277 between ca. 32–27 Ma (Pascher et al., 2015; Figure 5) as well as the reduced presence of eutrophic‐preference silicoflagellate *Bachmannocena* after the EOT (McCartney et al., 2023). Several previous studies have similarly linked intervals of elevated nutrients to larger calcareous nannoplankton (cell) size (Guitián, Jones, et al., 2020; Ma, Aubry, et al., 2023; Ma, Jin, et al., 2023). Others have invoked shifts in thermal gradients, water column structure, and nutrient availability through the greenhouse‐icehouse transitions as a key driver of macroevolutionary trends in diatom and dinoflagellate size through the Cenozoic (Finkel et al., 2005, 2007).

As well as playing an important role in the size structure and composition of phytoplankton communities, nutrient availability stimulates primary production and is a critical regulating factor in phytoplankton standing stocks, that is, cells mL^−1^ (e.g., Chavez et al., 2011). Our analysis does not attempt to systematically scale our results to nutrient‐driven variability in phytoplankton standing stocks, although this may be feasible in future if high resolution paleoproductivity proxies become available. Instead we assumed a fixed total abundance of 100 cells mL^−1^ through the record (see Methods). However, we can explore the relative impact of changes in size structure on “total community” POC and PIC using some simple, hypothetical assumptions of the relative change in standing stocks that might occur between the inferred interval of enhanced nutrient availability in the latest Eocene‐earliest Oligocene relative to the mid‐late Oligocene. If we assume that increased nutrient availability between the latest Eocene and the earliest Oligocene hypothetically increased standing stocks by 10% (i.e., from 100 cells mL^−1^ at 34.4 Ma to 110 cells mL^−1^ at 33.5 Ma), the increase in cells mL^−1^ combined with the increased size diversity and greater presence of larger cells represented by earliest Oligocene community composition would result in a 54% increase in total community POC (from 9,261 pg C mL^−1^ at the onset of the EOT to 14,308 pg C mL^−1^ immediately after the EOT) and a 57% increase in total community PIC (from 13,975 to 21,949 pg C mL^−1^) compared to the latest Eocene. Later in the Oligocene when nutrient availability and therefore standing stocks may have been lower than in the earliest Oligocene, the shift in community size structure towards smaller size classes would likely be sufficient to sustain a similar or even greater abundance of cells relative to the earliest Oligocene. Continuing with our example of 110 cells mL^−1^ in the earliest Oligocene (ca. 33.5 Ma), the dominance of small cells by the Late Oligocene (ca. 26 Ma) would have sustained a higher abundance of nannoplankton at the site (up to 235 cells mL^−1^, an increased abundance of 114%), even if the carrying capacity of the community decreased by 20% to a total community POC of 11,450 pg C mL^−1^. Based on these simplified scenarios, it is clear that shifts in community size structure that are coupled with, or decoupled from, changes in nutrient availability influence the total organic and inorganic carbon production of the community as well as how that carbon is partitioned across size classes (e.g., Figure 4).

In addition to nutrient availability, long‐term changes in atmospheric CO_2_ concentrations through the Cenozoic have also been proposed as a factor influencing plankton size evolution (Finkel et al., 2005, 2007; Henderiks & Pagani, 2008; Schmidt et al., 2004). Several studies link the evolution of calcareous nannoplankton size and calcification to the significant decline in CO_2_ levels through the Cenozoic, notably the evolution from many large, heavily calcified species in the Eocene to common small, less heavily calcified species by the late Neogene that is particularly evident within the Noelaerhabdaceae (Bolton et al., 2016; Claxton et al., 2022; Guitián, Jones, et al., 2020; Hannisdal et al., 2012; Henderiks & Pagani, 2008; Henderiks & Rickaby, 2007; Ma, Aubry, et al., 2023; McClelland et al., 2016; Šupraha & Henderiks, 2020). Laboratory studies indicate that some, but not all, extant coccolithophore species/strains have smaller cell size under lower CO_2_ concentrations (e.g., de Bodt et al., 2010; Le Gueval et al., 2024; Müller et al., 2015; Olson et al., 2017; Rickaby et al., 2016). This may be because smaller cells are more competitive at diffusive gas uptake, have growth optima at low CO_2_ concentrations, and an overall lower cellular carbon demand (small cells, lower cellular POC, less heavily calcified) (e.g., Chauhan & Rickaby, 2024). However, we note that the cellular carbon demand of Noelaerhabdaceae and Coccolithaceae species during the Eocene‐Oligocene was likely more similar than is the case for extant representatives of these families, as Oligocene *Reticulofenestra* and *Cyclicargolithus* exhibit a greater size range and are generally more heavily calcified than their modern descendants (PIC:POC ca. 0.8–2.8 compared to ca. 0.5 and 1.1 for *Emiliania* *huxleyi* and *Gephyrocapsa oceanica*, respectively; McClelland et al., 2017). Nevertheless, it is feasible that declining *p*CO_2_ levels across the Eocene and Oligocene exerted additional selective pressure on large taxa, contributing to the loss of the largest reticulofenestrid species in the early Oligocene and contributing to the shift in community size structure at Site U1553 towards smaller size classes. In addition, trade‐offs between size, inter‐specific differences in carbon demand (e.g., Chauhan & Rickaby, 2024), potential active carbon uptake mechanisms (e.g., Mackinder et al., 2010; Rokitta & Rost, 2012), and nutrient half saturation constants (e.g., Flynn et al., 2018; Smith et al., 2014) across different lineages may contribute to the greater variability in size diversity observed in the Noelaerhabdaceae (Figure S9 in Supporting Information S1; Young, 1990) compared to other lineages.

### 4.3 Implications for the Biological Carbon Pump

Changes in the composition and size structure of planktonic communities influences the efficiency of the biological carbon pump and the flux rate of POC from the surface to the deep ocean (Bopp et al., 2005; Guidi et al., 2008, 2016; Henson et al., 2022; Laufkötter et al., 2016; Serra‐Pompei et al., 2022; Stukel et al., 2024) by impacting organic matter sinking speeds, formation of aggregates, and the size and sinking rates of zooplankton fecal pellets (De La Rocha & Passow, 2007; Sanders et al., 2010; Schmidt et al., 2014). Models suggest that shifting phytoplankton communities toward smaller size classes (and possibly altering community composition by favoring some groups over others) may reduce the size and sinking speed of particulate matter, thereby decreasing POC export (Henson et al., 2022) and leading to a weaker biological carbon pump. On the other hand, the slower sinking speeds of smaller particles may increase nutrient recycling at shallower water column depths and faster recirculation of nutrients into the photic zone, enhancing surface productivity and offsetting some of the decline in POC export that might be expected from smaller particle sizes (Leung et al., 2021).

Using these modeling results as a simplified framework to summarize interactions between community size structure and the biological carbon (soft tissue) pump, the shift from larger mean size and broader size diversity in the latest Eocene‐earliest Oligocene to smaller cell sizes for the remainder of the Oligocene may indicate that nannoplankton‐derived POC had greater export potential across the EOT and earliest Oligocene and comparatively reduced potential for export for the remainder of the Oligocene. Lower community PIC:POC in the earliest Oligocene may have moderated the impact of the larger cell sizes on POC export by dampening nannoplankton sinking speeds and the ballasting of exported organic matter (Barker et al., 2003; Biermann & Engel, 2010; Rosas‐Navarro et al., 2018), although we note that community PIC:POC (Figure 3) and the PIC:POC of all but the smallest size classes (Figure 4) remained >1 throughout the record.

Biogenic calcification and vertical carbonate fluxes (the carbonate pump) also impact global CaCO_3_ burial and seawater alkalinity (e.g., Boudreau et al., 2018), as the production of 1 mol of CaCO_3_ removes 2 mol of alkalinity and 1 mol of DIC from seawater (Sarmiento & Gruber, 2006). The consumption of seawater alkalinity in turn reduces the CO_2_ buffering capacity of surface waters, regionally in areas with a high abundance of calcifying organisms and globally via alkalinity transfer to intermediate and deep‐waters that transport alkalinity to other regions (Krumhardt et al., 2020; Planchat et al., 2023). Fluctuations in the productivity, calcification and the PIC:POC ratio of pelagic calcifiers therefore affect biological pump efficiency (De La Rocha & Passow, 2007; Guerreiro et al., 2021), ocean carbon storage (Matsumoto et al., 2002) and the air‐sea partitioning of carbon (Archer & Maier‐Reimer, 1994; Bolton et al., 2016; McClelland et al., 2016) and are plausibly of sufficient magnitude to contribute to glacial‐deglacial fluctuations in atmospheric CO_2_ (Archer & Maier‐Reimer, 1994; Munhoven, 2007; Rickaby et al., 2010). At Site U1553, community PIC:POC steadily rises through the Early Oligocene (from 1.5 to 1.9) and then remains broadly consistent through the mid‐late Oligocene (Figure 3). If this trend toward higher community PIC:POC at Site U1553 is indicative of enhanced calcification across the region, increased PIC:POC in the mid‐late Oligocene would have consumed more seawater alkalinity, especially as nannoplankton community PIC:POC approached the critical theoretical value of 1.86 where calcification completely counteracts photosynthesis (McClelland et al., 2016).

To fully understand how nutrient utilization, export productivity and calcification interact to influence the biological carbon pump, we would additionally require information on rates of POC and PIC production by the community through time and community standing stocks (cells mL^−1^) that cannot be inferred from our data sets. Species with low POC (PIC) content can be significant producers of POC (PIC) in a community if they are both abundant and able to sustain much higher growth rates compared to species with larger cellular POC (PIC) under the same environmental conditions. In present‐day subarctic North Atlantic communities, for example, the small and lightly calcified *E. huxleyi* can only dominate community calcite production if species with high cellular PIC (namely *Coccolithus pelagicus* at these latitudes, 30× greater PIC than *E*. *huxleyi*) have vastly lower growth rates (a comparative growth rate <15% *E*. *huxleyi*) or when high PIC species are present in very low abundances (Daniels et al., 2014, 2016). It is therefore possible that species present in low abundances at Site U1553 but with a disproportionately large contribution to community POC and/or PIC due to their large size or high PIC:POC (e.g., *Z*. *bijugatus*) could have been (more) considerable producers of biomass or calcite in the community if they were able to maintain higher growth rates than other taxa in the community.

Given the range of cellular PIC:POC across both common and rarer morphogroups present at Site U1553 (Figure 2; Figure S9 in Supporting Information S1), changes in community composition through time drives the ca. 25% increase in community PIC:POC we observe through the early Oligocene (Figure 3). Gibbs et al. (2018) reported similar magnitude increases (23%–26%) in community PIC:POC across the Paleocene‐Eocene Thermal Maximum (PETM), although the absolute mean PIC:POC of those PETM communities were substantially lower (0.85–1.15) than we observe for the Oligocene (1.53–1.90). Increases in community PIC:POC of this magnitude could be partially or fully offset by differences in productivity between species or by differences in productivity between calcareous nannoplankton and other calcifying and non‐calcifying plankton groups. Until we can satisfactorily resolve nannoplankton calcification rates and changes in the productivity of nannoplankton relative to other plankton groups, we cannot fully resolve the consequences of changes of calcareous nannoplankton size structure and community biogeochemical traits on the biological carbon pump. This could be addressed in future by combining assemblage records for other plankton groups with records of nutrient availability, productivity, and ocean chemistry at Site U1553.

## 5 Conclusions

The diversity of calcareous nannoplankton size and morphological traits directly influences the organic and inorganic carbon content (cellular POC and PIC, respectively) of calcareous nannoplankton species and subsequently impacts the contribution of different species to community biogeochemistry. Using data sets of relative abundance and morphometry, we reveal the first cellular POC and PIC estimates for Oligocene nannoplankton and reconstruct a timeseries of community size structure and associated community biogeochemical traits (POC, PIC, and PIC:POC) between the latest Eocene and the mid‐late Oligocene at high latitude South Pacific Ocean Site U1553. The community is dominated by temperate‐affinity taxa throughout but changes in relative species abundance across the EOT and during the Oligocene shifted community structure away from a size diverse, earliest Oligocene community to a mid‐late Oligocene community dominated by smaller species and higher overall PIC:POC. We propose that these changes in community composition and community biogeochemical traits through the Oligocene are linked to evidence for increased nutrient availability at Site U1553 and regionally between the late Eocene and early Oligocene. Whilst the taxa present at Site U1553 exhibit a range of cell sizes and cellular biogeochemical traits, the most common species present in mid‐late Oligocene assemblages have high cellular PIC:POC that contributes to increasing community PIC:POC through the Oligocene. Our results demonstrate that changes in community composition will have greater biogeochemical consequences when species' biogeochemical traits are distinctive from the rest of the community (e.g., increased/decreased abundance of species with very high PIC:POC or large cell size). This emphasizes the need to understand the diversity of nannoplankton size and morphological traits to better assess the contribution of different species to community biogeochemistry and for understanding the broader biogeochemical impact of calcareous nannoplankton responses to past (and future) climate change.

## Supporting information

Supporting Information S1

## Data Availability

This research used samples provided by the International Ocean Discovery Program (IODP). Calcareous nannoplankton primary data sets generated for this study (assemblage relative abundance and coccolith morphometrics) are available at zenodo as Sheward, Herrle & Fuchs (2024), doi: 10.5281/zenodo.11472764. The Eocene‐Oligocene fossil coccosphere geometry data set is available at zenodo as Sheward, Gibbs, et al. (2024), doi: 10.5281/zenodo.11473074. IODP Expedition 378 shipboard data are available via https://web.iodp.tamu.edu/OVERVIEW/.
