# Supplementary material for: Biogeochemical Traits of a High Latitude South Pacific Ocean Calcareous Nannoplankton Community During the Oligocene
Source: Paleoceanogr Paleoclimatol. 2024 Nov 28;39(12):e2024PA004946. doi: 10.1029/2024PA004946 (PMC11604600; doi:10.1029/2024PA004946)
Supplement: Supplementary file 1 — Supporting Information S1 [file PALO-39-0-s001.pdf]

Paleoceanography and Paleoceanography

Supporting Information for

Biogeochemical traits of a high latitude South Pacific Ocean calcareous nannoplankton community during the Oligocene

Rosie M. Sheward[1], Jens O. Herrle[1], Julian Fuchs[1], Samantha J. Gibbs[2], Paul R. Bown[2], and Pia M. Eibes[3]

[1]Institute for Geosciences, Goethe-University Frankfurt, Frankfurt am Main, Germany.

[2]Department of Earth Science, University College London, London, UK.

[3]Insitute of Physical Geography, Goethe-University Frankfurt, Frankfurt am Main, Germany.

Contents of this file

Text S1 to S4
Figures S1 to S10
Table S1

Introduction

Text S1 provides further information about the IODP Site U1553 age model used in this study and calcareous nannoplankton biostratigraphic markers identified in our samples used to inform the age model.
Text S2 to S4 provide additional methodological considerations for the reconstruction of cell size, cellular particulate organic carbon (POC) and cellular particulate inorganic carbon (PIC).

Figure S1 is a map of the location of IODP Site U1553 during the Oligocene.
Figure S2 is a plate illustrating fossil coccospheres for the morphogroups used in this study.
Figures S3 to S7 are supporting figures for detailed aspects of the methodology.
Figures S8 to S10 show further detailed results that support the findings and interpretation of this study (e.g., morphogroup-specific biogeochemical traits through time).

Table S1 presents the age and mid-point depth tie points for the age model.

Text S1. Site U1553 age model and calcareous nannoplankton biostratigraphic events

The initial shipboard age model (Röhl et al., 2022) was based predominantly on age-depth tie-points for calcareous nannofossil and planktonic foraminifera bioevents at ca. 10 m depth resolution following the Southern Ocean biozonation scheme of Fioroni et al. (2012) for calcareous nannoplankton and the biozonation scheme of Huber & Quillévéré (2005) for planktonic foraminifera. Identified bioevents are described as "base" (B) for the stratigraphically lowest occurrence of a taxa or "top" (T) for the stratigraphically highest occurrence of a taxa. "Base/Top common" (Bc/Tc) refers to the first/last continuous and common (ca. >0.5% abundance) occurrence of a taxa, respectively, and "Base increase" (Bi) is used to define the start of an interval of increased abundance (acme). We have refined six of the calcareous nannofossil depth tie-points reported in Röhl et al. (2022) based on our observations. The three planktonic foraminifera bioevents are based on shipboard core catcher samples and the depth of these bioevents have been estimated based on the depth of the bioevent in each Hole relative to the splice interval tables of Drury et al. (2022). The biostratigraphic age-depth tie points used for the age model in this study are shown in Table S1 relative to the Geological Time Scale (GTS) 2012 (Gradstein et al., 2012). Sample ages are calculated assuming a linear sedimentation rate (ranging between approx. 1.5 and 3.2 cm/ky; Röhl et al., 2022) between mid-point depth tie-points.

Based on the identified bioevents (Table S1), the studied section spans the Late Eocene *Reticulofenestra oamaruensis* Zone (37.7 Ma) to the Late Oligocene *Chiasmolithus altus* Zone (ca. 26.5 Ma). The presence of *R. oamaruensis* in our lowest sample (206.16 m-CCSF) indicates that our record begins in the late Eocene *R. oamaruensis* Zone. The T *R. oamaruensis* is clearly identified in our samples at 197.12 m-CCSF, defining the top of this Zone and used to denote the approximate position of the Eocene-Oligocene Boundary (EOB) following Fioroni et al. (2012). In mid- and low latitude sections, the base of the Eocene-Oligocene Transition (EOT) is approximately coincident with T *Discoaster saipanensis* (Agnini et al., 2014) but this event has strong latitudinal diachrony and, as discoasters are generally rare and intermittently present in our U1553 samples, this bioevent was not identified in our samples. The B *Chiasmolithus altus* bioevent for the early Oligocene B *Blackites spinosus* Zone could not be confidently identified from our samples, as the central area structure of *Chiasmolithus* coccoliths were often damaged, lost or overgrown thus preventing confident species-level identification, especially for specimens with intermediate morphology between *Ch. altus* and *Chiasmolithus oamaruensis*. However, clear specimens of *Ch. altus* were consistently present (>0.5 %) from 186.9 m-CCSF (Core 378-U1553E-20X) and we use as a tentative depth for the base of the *Blackites spinosus* Zone. We identified T *Ismolithus recurvus* in our samples at 166.06 m-CCSF (defining B *Reticulofenestra daviesii* Zone). The Tc *Reticulofenestra umbilicus* (where relative abundance fell below 0.5 %) was recorded in our samples at 150.86 m-CCSF, marking the B *Ch. altus* Zone (T *R. umbilicus* was difficult to define due to several scattered occurrences in later Oligocene samples). The B *Reticulofenestra/Dictyococcites bisecta* Zone (defined by the T *Ch. altus*) was not observed, as *Ch. altus* was present in all Late Oligocene samples examined.

Text S2. Modelling cell size

Coccolith size has been used as a proxy for cell size (e.g., Henderiks and Pagani, 2007) but there is significant natural variability in the relationship between coccolith and cell size due to a wide range of species-specific coccosphere architectures (Bown et al., 2014; Gibbs et al., 2013, 2018) that influence the number of coccoliths per cell, C_N, which can range from 5 to several hundred per cell depending on the species (e.g., Sheward & Poulton, 2024).

The cell size distribution of each morphogroup was modelled following a previously published cell size model (Gibbs et al., 2018). The input for the model are morphogroup-specific coccosphere geometry relationships (the relationship between cell size, coccolith size and number of coccoliths per cell) measured from intact fossil coccospheres (Figure S3) and datasets of the frequency distribution of coccolith size (C_L) and number of coccoliths per cell (C_N) in each sample. For each sample, the method to model the cell size distribution of each morphogroup proceeded as follows (see also Gibbs et al., 2018; an overview of the method for placolith groups is shown in Figure S6):

Step (1) for placolith morphogroups, the coccolith size-cell size relationship was derived from fossil coccosphere geometry data (Equation 1, main text; Figure S3);

Step (2) the frequency distribution of C_N was analysed from the (subset) of fossil coccosphere geometry data (bin size = 1) for placolith taxa. For *Discoaster*, *Sphenolithus* and *Z. bijugatus*, a Gaussian-like C_N distribution was alternatively simulated (Text S3, Figure S5);

Step (3) the frequency distribution of C_L (bin size = 0.5 µm) was analysed for each morphogroup from sample-specific loose coccolith measurements;

Step (4) morphogroup cell size was calculated using Equation (1) or Equation (3) (for *Discoaster*, *Sphenolithus* and *Z. bijugatus*) for all possible combinations of C_N and C_L. A maximum of 3,250 possible C_L-C_N combinations were considered, where C_N ranged from 1 to 65 and C_L ranged from 0 to 26 µm;

Step (5) the likelihood of each C_L-C_N combination occurring in the sample was calculated, based on the frequency distribution of C_L (sample-specific) and C_N (morphogroup specific). 'Likelihood' is calculated by applying the frequency distribution of C_N to each C_L size bin. For example, 60 of the coccoliths in the sample are 7.5-8.0 µm. We know from the frequency histogram of C_N that for every C_L size class, e.g., 10 % of cells have 12 coccoliths per cell, 20 % of cells have 14 coccoliths per cell, 14 % of cells have 15 coccoliths per cell, etc. Therefore, 10% of the coccoliths 7.5-8.0 µm (6 coccoliths) are associated with a cell with a C_N of 12 thus representing 0.5 cells, 20% of the coccoliths 7.5-8.0 µm (12 coccoliths) are associated with a cell with a C_N of 14 thus representing 0.86 cells, and so on for all combinations of C_L and C_N.

Step (6) the results of Step (4) and Step (5) were summed pairwise for each C_L-C_N combination to generate a frequency distribution of cell size in the morphogroup that was then scaled to the relative cellular abundance of the morphogroup in the sample (first converting relative abundance from coccolith counts into relative cellular abundances using a mean morphogroup C_N; Table 2). To smooth out any large peaks and troughs in relative abundance between consecutive samples, we used a 5-point moving average of the percentage relative coccolith abundance dataset (calculated using the 'smoothing spline' function in PAST4; Hammer et al., 2001) as the abundance weighting factor for each morphogroup. We assume that every sample represents a community of 100 cells, i.e., we have not further scaled the community to different population carrying capacities.

Text S3. Coccosphere geometry reconstructions for *Discoaster* spp., *Sphenolithus* spp., and *Zygrhablithus bijugatus*

Whilst *Discoaster*, *Sphenolithus* and *Z. bijugatus* are present in the Oligocene assemblage at Site U1553 in only low abundances (up to ca. 2 %) it is important to estimate the contribution of these species to community particulate organic and inorganic carbon (POC and PIC, respectively) in case 'rare' species contribute disproportionately to biomass and/or calcite relative to their abundance.

The cell size and size-trait model (Methods and Text S2) requires two input datasets to model morphogroup cell size: (1) a frequency histogram of C_L of the morphogroup in the sample (or samples of similar age) and (2) a frequency histogram of C_N on coccospheres of the morphogroup. Poor preservation potential means that we lack coccosphere geometry data from intact fossil coccospheres for *Discoaster*, *Sphenolithus* and *Z. bijugatus* and therefore cannot derive Equation (1) from intact coccospheres (see Methods) for these morphogroups and do not have an input frequency histogram of morphogroup C_N.

To overcome these two challenges, we simulated a hypothetical C_N distribution for these morphogroups that can be used in place of directly measured morphogroup C_N distribution from fossil coccospheres to use as input in the size-trait model and to estimate morphogroup size-specific cellular PIC. We estimate cell size for each C_L-C_N combination based on an alternative parameterisation of the relationship between cell size, coccolith size and C_N (Equation 3, Methods) This new method can also be applied to other non-placolith taxa with no intact fossil coccosphere observations, providing that some observational information concerning a reasonable C_N range is available, for example, from collapsed coccospheres or modern analogue species.

Simulated morphogroup C_N distribution. For each morphogroup, we simulated the frequency distribution of C_N as follows:

1. A 'mean' C_N for the morphogroup was estimated informed by the C_N observed on extant species with coccoliths/nannoliths/holococcoliths of broadly similar morphological characteristics and the C_N range given in Gibbs et al. (2018) based on observations of the C_N of rare collapsed fossil coccospheres and constraints posed on cell size suggested by lith curvature.

2. The standard deviation (SD) of the mean C_N was estimated, informed by the mean C_N and SD of extant coccolithophore species with similar C_N ranges (if roughly known for fossil taxa) and similar lith shape/size. Two SDs were estimated: an average SD that is typically for the C_N of extant and fossil species with similar C_N and a larger SD that aims to generate C_N values that aims to capture the occurrence of relatively few, larger coccospheres with higher C_N that are observed in the C_N distribution of extant species in culture (Gibbs et al., 2013; Sheward et al., 2017).

3. Using this mean and SD, we generated ten random C_N 'datasets' (n=113) that follow a normal distribution using the NORMINV function in Excel (=NORMINV(RAND(), mean, SD)). This simulated C_N dataset consists of 63% data (n=83) generated using the average SD and 37% data (n=31) generated using the larger SD. The simulated C_N frequency distribution for the morphogroup was then the average of the frequency distribution (histogram with size bin = 1) of each artificial C_N dataset (Figure S5).

4. The simulated C_N frequency histogram was used as the data input to estimate the probability of each C_L-C_N combination occurring in coccospheres of the morphogroup, the cell size of each C_L-C_N combination (using the alternative approach detailed below), and the cellular PIC of each C_L-C_N combination (as described in the Methods).

Alternative coccosphere geometry relationships. We developed an adjusted method to estimate the cell size of each possible C_L-C_N combination based on the logic that the surface area of the cell ($\Theta_{SA} = 4\pi r^2$) is related to cell diameter ($\Theta = 2r$) and can be described as a function of the surface area of the (proximal) coccolith surface (C_{SA}) and C_N (i.e., the sum of coccolith surface areas), with consideration for the degree of either overlap (placolith and placolith-like coccoliths) or spaces (e.g., muroliths and holococcoliths) between the adjacent proximal surface of each coccoliths on the cell surface that respectively subtract from or add to cell surface area relative to the sum of coccolith surface areas.

Conceptually, this approach draws on a mathematical geometry problem called "circle packing on a sphere" and explores the maximum size, *d*, of *N* circles of identical size packed onto the surface of a sphere of radius *r* without any overlap (e.g., Clare and Kepert, 1991, 1986). The packing density, *p*, is the fraction of the spherical surface enclosed by the circles and mathematically cannot exceed 0.906900, i.e., up to 90.69 % of the spherical surface can be enclosed by *N* circles of size *d* (*p* cannot exceed this value for a close packed plane as *N* approaches infinity (Clare and Kepert, 1991). For $N = 2$ to 40, solutions for *p* range between 0.732233 and 0.89095 (Clare and Kepert, 1986). For $N = 40$ to 80, solutions for *p* range between 0.83 and 0.85 (Clare and Kepert, 1991). If we consider that *N* is equivalent to the C_N of a coccosphere formed of circular liths of identical proximal surface size (C_{LP}), then we can assume that for $C_N = 5$ to 80, at least 73.22 % to 89.60 % of the cell surface area can be covered by the surface area of the liths and therefore at least 10.40 to 26.78 % of cell surface area must be represented by gaps between adjacent, abutting coccoliths (Figure S4).

The packing of ellipses on a sphere (i.e., sub-circular to elliptical coccoliths with an aspect ratio >1) is mathematically more complex, as more options exist for ellipse arrangement on a spherical surface, which is also related to the aspect ratio of the ellipse. However, *p* for ellipses on a spherical surface is generally higher than *p* for circles (for the same *N* and aspect ratio) and *p* for ellipses with an aspect ratio ca. >1.2 tends to be ca. >0.86, especially when $N>35$ (Gnidovec et al., 2022). We can therefore assume that ca. 86-88% of the cell surface area of species with elliptical coccoliths would be covered by coccolith surface area and that this is relatively consistent as C_N increases above $C_N = 35$. The generally greater *p* of ellipses compared to circles for the same *N* (Gnidovec et al., 2022) also implies that that a species with elliptical liths (lith bases) would likely surround a cell with smaller equivalent cell diameter than a species with the same number of circular or sub-circular liths.

We define a new term, C_O (in Equation 3, Methods), referring to the fraction of cell surface area that is covered by overlapping adjacent coccoliths (e.g., placolith and planolith morphologies) or the fraction of cell surface area that is associated with gaps between abutting but not overlapping coccoliths (e.g., murolith and holococcolith morphologies). An overlap <1 indicates an overlapping coccolith arrangement, which reduces the surface area of the reconstructed coccosphere relative to the total surface area of all coccoliths forming the coccosphere (i.e., $C_O = 0.8$ reduces cell surface area by 20%). An overlap of >1 increases the surface area of the reconstructed coccosphere relative to the total surface area of all the coccoliths forming the coccosphere to account for the additional surface area represented by

gaps between adjacent coccoliths. For circular, non-overlapping coccoliths, C_O must exceed 1.11 (i.e. 11% additional surface area for gaps) and for C_N of 5-80, C_O is likely to range between 1.15 and 1.27 (15 to 27% additional surface are for gaps) based on the mathematical theory of circle packing on a sphere. The aspect ratio (*AR*) was determined from lith measurements and overlap percentages (C_O) for each morphogroup were estimated from coccospheres of extant species with broadly similar coccolith morphological characteristics, as described in the following taxonomic considerations:

Considerations for *Z. bijugatus* coccosphere reconstructions. *Z. bijugatus* is a robust fossil holococcolithophore that is generally common in Paleogene assemblages with an elliptical base shape that rises into a cruciform rod of variable height (ca. 5 to 16 µm; Nannotax3 website, 2023). Despite the wide diversity of extant holococcolith morphologies, no species have directly comparable morphology to *Z. bijugatus*, although some have holococcoliths with process-like features (e.g., *Calicasphaera diconstricta, Flosculosphaera* spp. and *Calyptrosphaera heimdaliae*). Extant holococcolithophore coccospheres tend to share common features of small holococcolith base sizes (ca. 0.4 to 3 µm) associated with comparatively large coccospheres (where coccosphere diameter not including process height is typically at least an order of magnitude larger than holococcolith base size) and coccospheres with a high to very high number of holococcoliths per cell (C_N is ca. 30 to >150; Sheward & Poulton, 2024; Nannotax3 website, 2023). Assuming that Paleogene holococcolithophore species also had similar coccosphere architecture, *Z. bijugatus* coccospheres are likely to have been large relative to holococcolith base size and have had a relatively high number of coccoliths per cell, which Gibbs et al. (2018) estimate to be ca. 32 holococcoliths per cell.

Here, we assume the mean C_N of 22 for *Z. bijugatus* (a smaller value than the mean C_N of 32 used by Gibbs et al., 2018) as the input in our normally-distributed C_N simulation and an average SD of 5. As extant holococcolithophore coccospheres generally have a large C_N range, a SD of 5 is likely to be somewhat conservative. For example, mean C_N for the holococcolithophore phase of the extant genus *Syracosphaera* (mean C_N = 67, C_N range = 42-150, n = 21) has a standard deviation of 27 (Sheward & Poulton, 2024). To adjust for the likelihood that at least some coccospheres of *Z. bijugatus* would have had a higher C_N than would be simulated in a normal distribution using a SD of 5, 37% of the simulated dataset is generated using a higher SD of 10. Our simulation of a normally-distributed C_N for *Z. bijugatus* gave a C_N range of 9 to 37.

For the calculation of cell size for each C_L-C_N combination, we assume that the base measurement of *Z. bijugatus* always represents C_L, i.e. the longer size axis measurement, and that the mean *AR* of *Z. bijugatus* holococcoliths is 1.6 (narrowly elliptical) based on base C_L and C_W measurements of specimens in plan view. Based on observations of the orientation of elliptical-shaped holococcoliths (e.g., *Calcidiscus leptoporus* HOL, *Calyprolithina multipora*, *Corisphaera* spp.) on the surface of extant holococcolithophores and mathematical solutions for ellipse packing on a sphere (where N=32 and AR = 1.6, p = 0.88; Gnidovec et al., 2022), we assume that the base of *Z. bijugatus* holococcoliths sat side-by-side on the coccosphere surface with gaps between adjacent coccoliths that account for a minimum additional ca. 12% of cell surface area. We therefore apply a C_O of 1.13 for *Z. bijugatus* in the calculation of Θ.

Considerations for *Sphenolithus* coccosphere reconstructions. The Sphenolithaceae are very diverse Family of conical-shaped nannoliths with radial symmetry and spines of varying height. We based our choice of mean C_N of 42.5 for *Sphenolithus* on the C_N range of 27-60

reported by Gibbs et al. (2018) for *Fasciculithus* and *Sphenolithus*, which was informed by the assumption that the shallow curvature of the proximal (base) surface indicated a large cell size relative to nannolith size (Gibbs et al., 2018), also hypothetically illustrated by Towe (1979). As Sphenolithaceae and *Z. bijugatus* liths both have a concial/spinose shape, we followed similar reasoning to *Z. bijugatus* (described above) to determine an average SD of 5 and a larger SD of 10 for the simulation of morphogroup C_N distribution. Our simulation of a normally-distributed C_N for *Sphenolithus* gave a C_N range of 25 to 60.

For the calculation of Θ in *Sphenolithus,* we assume that the proximal surface of nannoliths are circular (*AR* = 1) and that gaps between the arrangement of nannoliths on the coccosphere surface would add a minimum additional 17% surface area to C_{SA} (here using C_O = 1.18) based on *p* for 42 circles packed tightly onto a sphere (Clare and Kepert, 1991) as there are no intact extant murolith- or holococcolith-bearing species with circular liths from which surface area measurements could be refined.

Considerations for *Discoaster* coccosphere reconstructions. *Discoaster deflandrei* (6-rayed stellate form) is the dominant *Discoaster* group of the Oligocene to Middle Miocene and also the most common species of *Discoaster* at Site U1553. We assigned a mean C_N of 20 for our *Discoaster* reconstructions with an average SD of 3 of a larger SD of 5. This reflects the C_N range of 20-31 inferred by Gibbs et al. (2018) for late Paleocene-early Eocene discoasters (namely rosette-shaped *Discoaster multiradiatus* and *Discoaster salisburgensis* that have a low degree of proximal surface curvature) based on the assumption that rosette-shaped nannoliths would likely completely cover the cell surface (with some nannolith overlap), similar to the genus *Calcidiscus* (a placolith taxa with relatively strongly overlapping coccoliths). The SD is based on the SD (=4) for the cellular C_N for extant *Calcidiscus* (Sheward et al., 2016). Our simulation of a normally-distributed C_N for *Discoaster* gave a C_N range of 10 to 31.

We viewed stellate discoasters as encompassed by a simple shape (i.e. a circle that encompasses the extent of all ray tips) and proceed as if we were considering a circular nannolith. We therefore assume a small degree of overlap as in Gibbs et al. (2018) for rosette-shaped species, i.e., comparable to the arrangement of rosette forms but with 'gaps' in coverage of the cell surface between ray elements. For the calculation of Θ based on slightly overlapping 'circular' nannoliths (*AR* = 1), we estimated the fraction of cell surface area covered by overlapping nannoliths to be 20% (i.e., the total surface area of 'circular' coccoliths is 120% of cell surface area, C_O = 0.8) based on the mean difference between total surface area of coccoliths (C_N x coccolith surface area) and coccosphere surface area of 29% for 1393 coccospheres of extant *Calcidiscus* (Sheward et al., 2016), which has more strongly overlapping coccoliths compared to our expectations for *Discoaster*.

Text S4. Shape factors in the estimation of coccolith calcite

Species-specific *Ks* values vary by approximately an order of magnitude (ca. 0.01 to 0.2) in extant coccolithophores (Young & Ziveri, 2000). We used published *Ks* values for extant *Coccolithus pelagicus* (*Ks* = 0.06) for *Coccolithus* as the fundamental morphology of *Coccolithus* changes little over the Cenozoic. Fewer *Ks* values have been published for extinct species, especially those of Eocene and Oligocene age. For *Chiasmolithus*, *Clausicoccus*, *Cyclicargolithus* and *Reticulofenestra* morphogroups, we adapted the *Ks* values of species with similar morphologies to adjust for features that likely increased or decreased the coccolith cross-sectional shape, following Gibbs et al. (2018). We use *Ks* values published by Preiss-Daimler et al. (2012) for *Sphenolithus abies* (mean *Ks* = 0.05) for cellular PIC estimates for *Sphenolithus* and the *Ks* value of 0.40 for *Z. bijugatus* from Agnini et al. (2016). For both morphogroups, C_L in the calculation of coccolith PIC (Equations 4 and 5, Methods) is the height of the nannolith or holococcolith, respectively. As *Discoaster deflandrei* is the most common *Discoaster* species in our assemblages, we adopted the *Ks* value of 0.22 published by Preiss-Daimler et al. (2012) for the Neogene species *Discoaster varibilis* as the morphology of *D. varibilis* is broadly similar to *D. deflanderi* (both 6-rayed stellate discoasters with bifurcated tips). As *D. varibilis* is slightly less massive and has a slightly concavo-convex cross-sectional shape, this *Ks* value may slightly underestimate nannolith PIC in our estimations. *Discoaster* PIC is calculated using ray length. The *Ks* values used here for *Sphenolithus* and *Discoaster* are in good agreement with mean *Ks* values reported by Guitián et al. (2022) for *Sphenolithus* (0.05, ranging between ca. 0.01 and 0.11) and *Discoaster* (0.02, ranging between 0.01 and 0.07) from Oligocene-age specimens from the North Atlantic Ocean based on birefringence-based thickness measurements. The *Ks* value used for each morphogroup are detailed in Table 2.

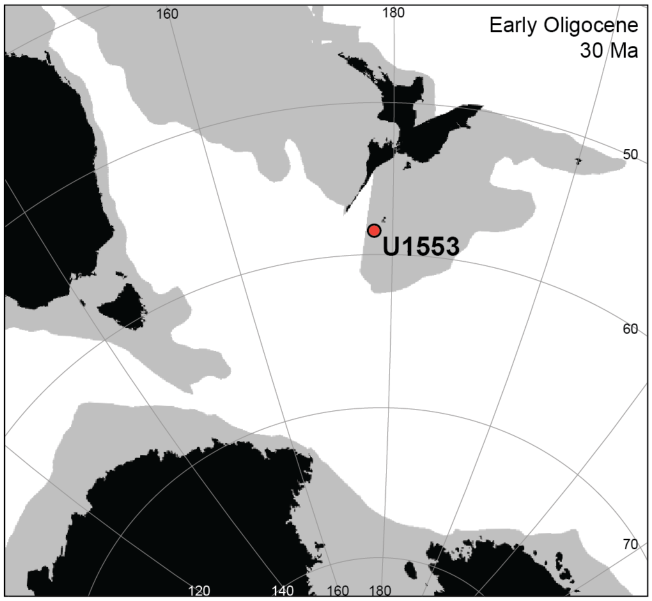

Figure S1. Map of the study site (IODP Site U1553) during the early Oligocene. The paleogeography at 30 Ma was reconstructed using GPlates (Müller et al., 2018; http://www.gplates.org; last accessed 7th March 2024) based on the global geodynamic rotation model of Müller et al. (2022). Land masses are shown in black and the continental shelf is shown in grey.

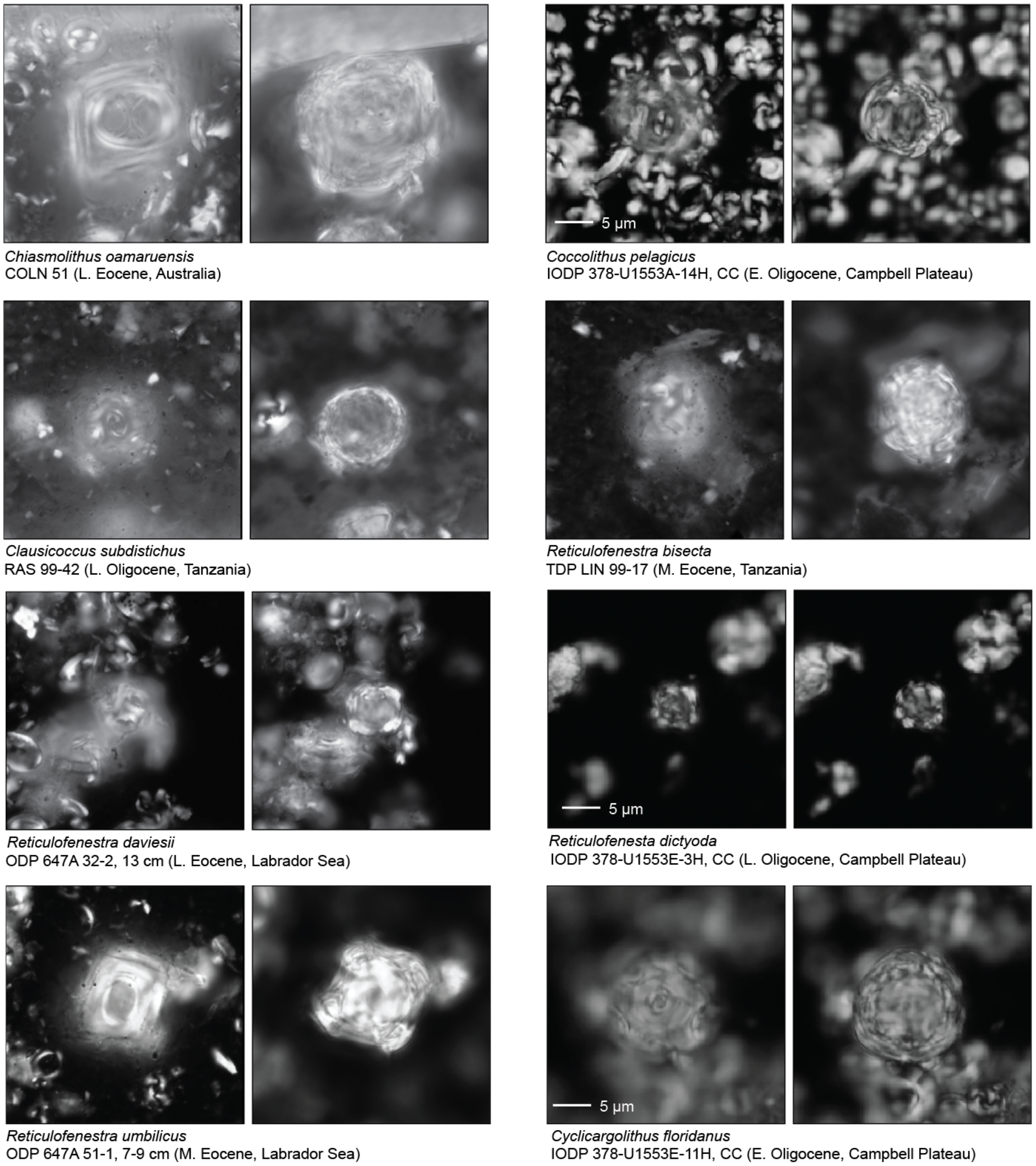

Figure S2. Intact fossil coccospheres imaged using polarised light microscopy. The left image of each pair is focused on the top surface of the coccosphere from which coccolith length can be measured. The right image of each pair is focused on the maximum diameter of the coccosphere (i.e. a cross-section through the coccosphere centre) from which coccosphere diameter (including the coccolith layer) and cell diameter (excluding the coccolith layer) can be measured. Examples are selected to show individuals from each of the morphogroups discussed in this study. Note that coccospheres of each species will exhibit a natural range of cell sizes and coccosphere geometries relating to the cell division cycle, growth environment, and genetic diversity. Information on the species name, sample reference, age and general locality are provided below each image. All images are to the same scale to illustrate the range in coccosphere sizes across different taxa.

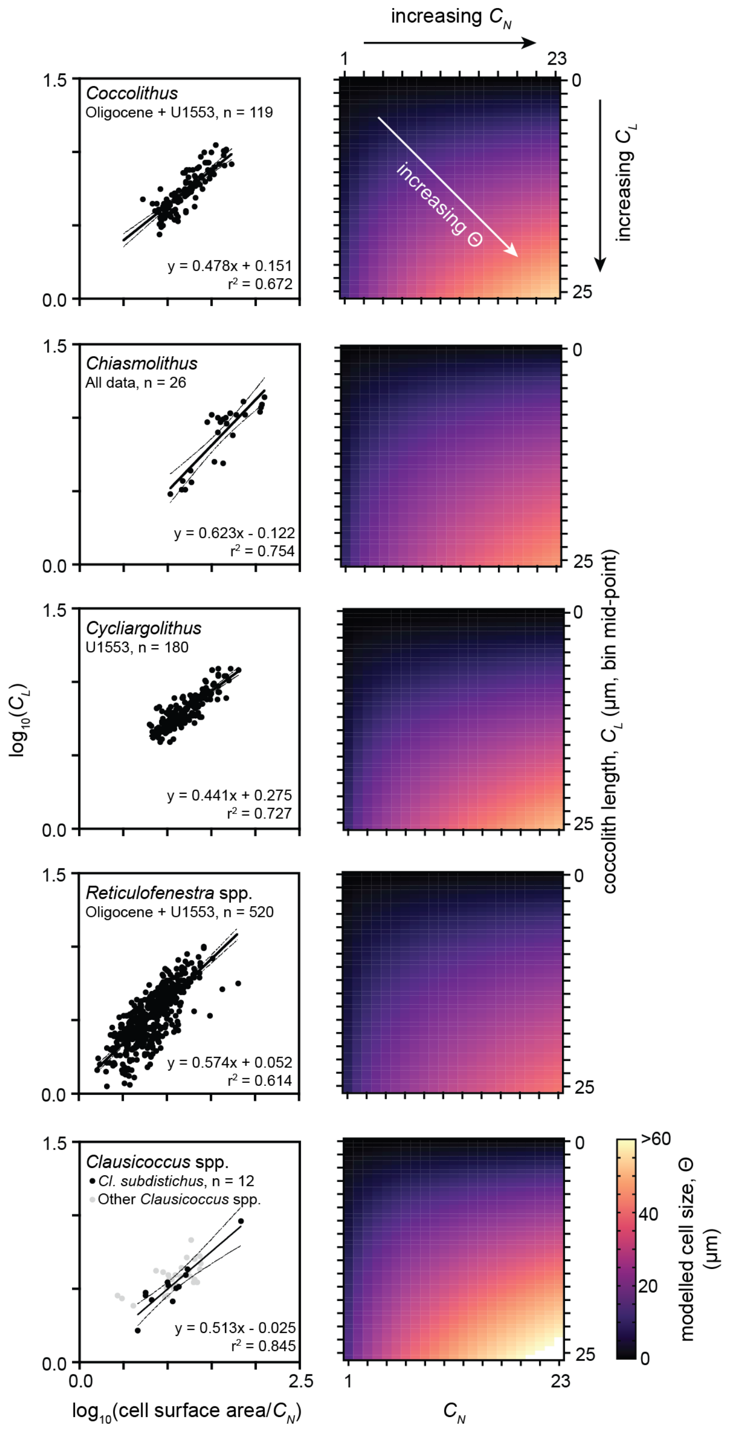

Figure S3. Coccosphere geometry relationship log-log plots with confidence intervals. Genus-specific constants α and β from the linear regression between $\log_{10}(C_L)$ and $\log_{10}$(surface area/C_N) are used in Equation (1). The heat map figures for each genus show the modelled cell size for any combination of C_L (0-25 µm) and C_N (1-23) using Equation (1) and is used in combination with measurement data of the C_L and C_N distribution of the morphogroup in each sample to calculate cell size histograms for each sample (see Methods and Text S3).

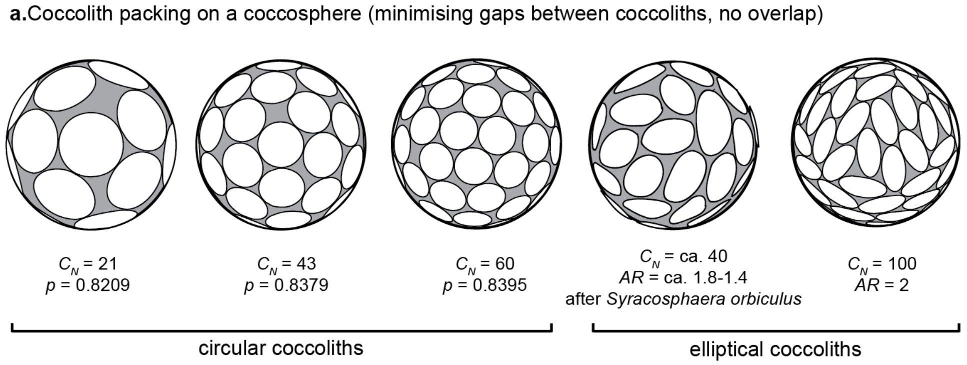

Figure S4. Example of mathematical circle and ellipse packing on a sphere, no overlaps, where p is packing density for varying numbers of coccoliths per cell, C_N. AR = aspect ratio of the ellipse. Illustrations are drawn after examples in Gnidovec et al., (2022) and Clare and Kepert, (1986, 1991).

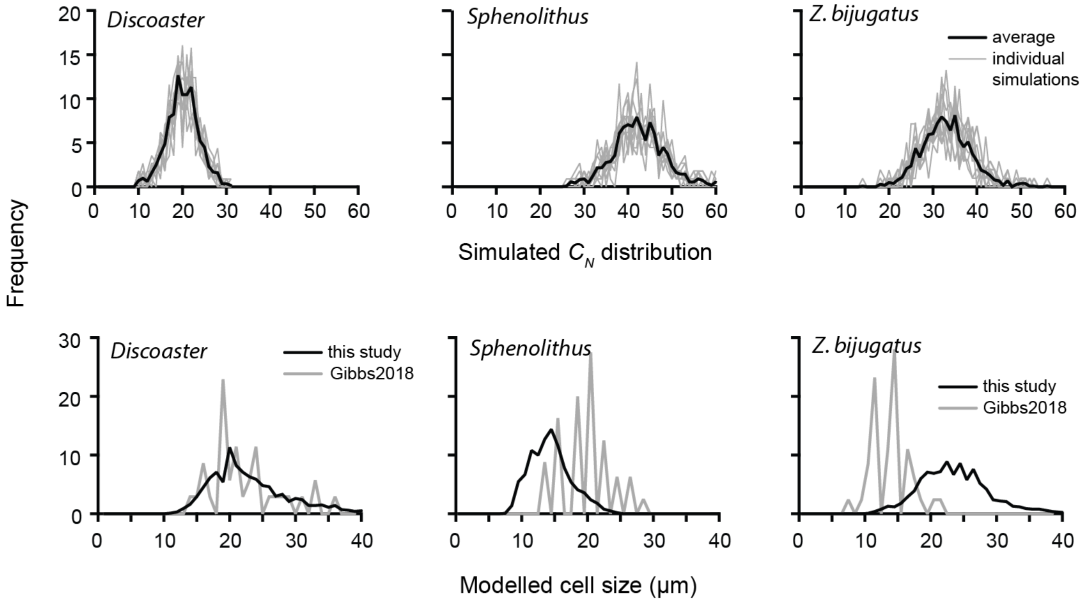

Figure S5. Simulated C_N distribution for *Discoaster*, *Sphenolithus* and *Z. bijugatus* morphogroups (top) showing the average C_N distribution (black) resulting from ten simulations (grey) and the resulting modelled cell size distribution (bottom, black line) based on this simulated C_N frequency distribution and the C_L frequency distribution measured from Site U1553 samples of latest Eocene and Oligocene age. For comparison is the modelled cell size that results from the linear C_L to cell size equations of Gibbs et al. (2018) for each morphogroup when used with the measured C_L histograms generated for our study (bottom, grey line).

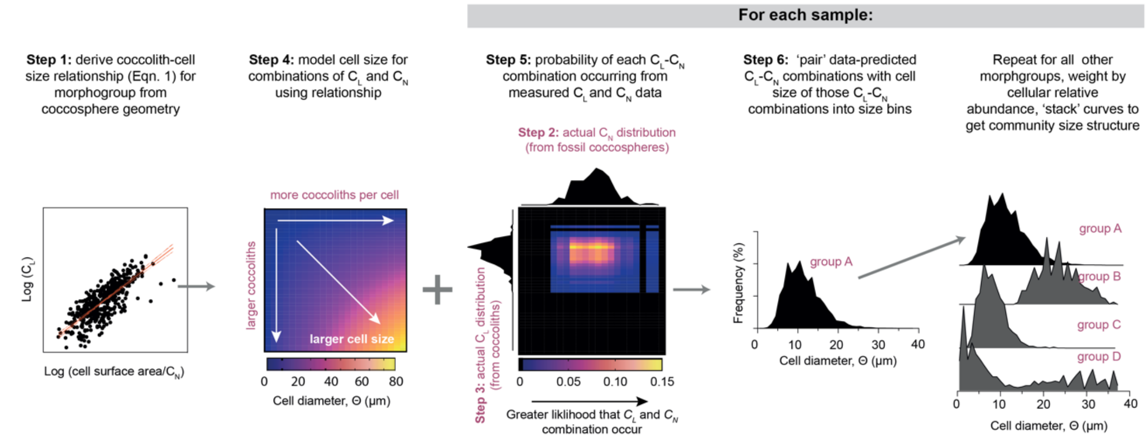

Figure S6. Methodological schematic of the model used to generate morphogroup cell size distributions in each sample, which are then weighted by cellular relative abundance and stacked to produce community cell size distributions. See Fig. S3 for Step 1 and Step 4 figures for *Chiasmolithus*, *Coccolithus*, *Cyclicargolithus*, *Reticulofenestra* and *Clausicoccus*.

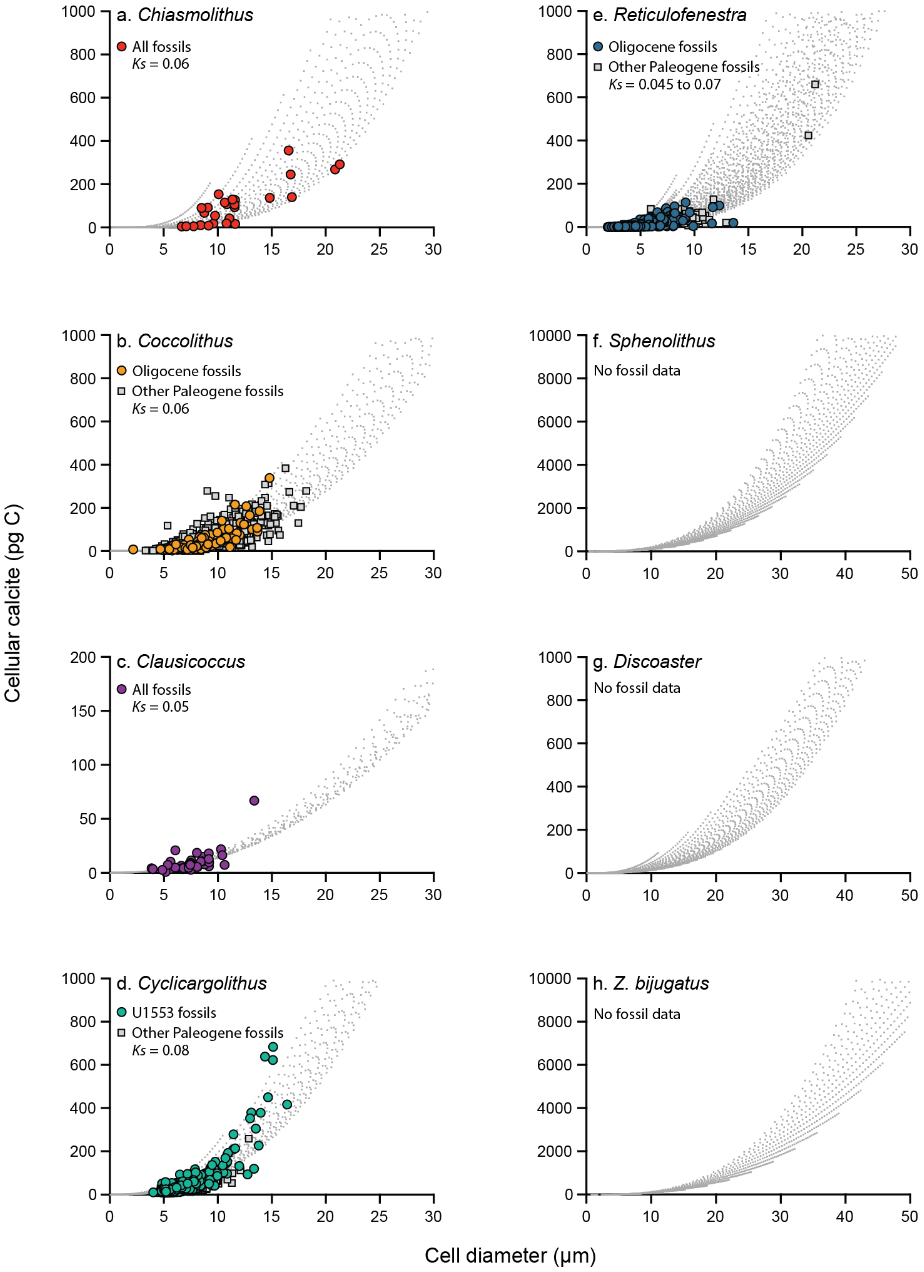

Figure S7. Cellular PIC as a function of cell size for each morphogroup. The range of cellular PIC resulting from the model (C_L-C_N pairs) are shown as small grey points. For context, the cell size and cellular PIC data from individual intact fossil coccosphere data used to inform the model (coloured points) are shown alongside other fossil coccosphere data from the Paleogene for *Coccolithus*, *Reticulofenestra* and *Cyclicargolithus* to show a wider range of fossil coccospheres observations (grey squares; Gibbs et al., 2013; Gibbs et al., 2018; Sheward, Gibbs, et al. 2024). *Reticulofenestra* morphogroups are pooled for the purpose of this figure. No fossil coccosphere data is shown for *Z. bijugatus*, *Sphenolithus* or *Discoaster* (f-h) as we do not have direct fossil coccosphere data for these taxa. The shape factor (*Ks*) used to calculate coccolith calcite is shown (see also Text S4).

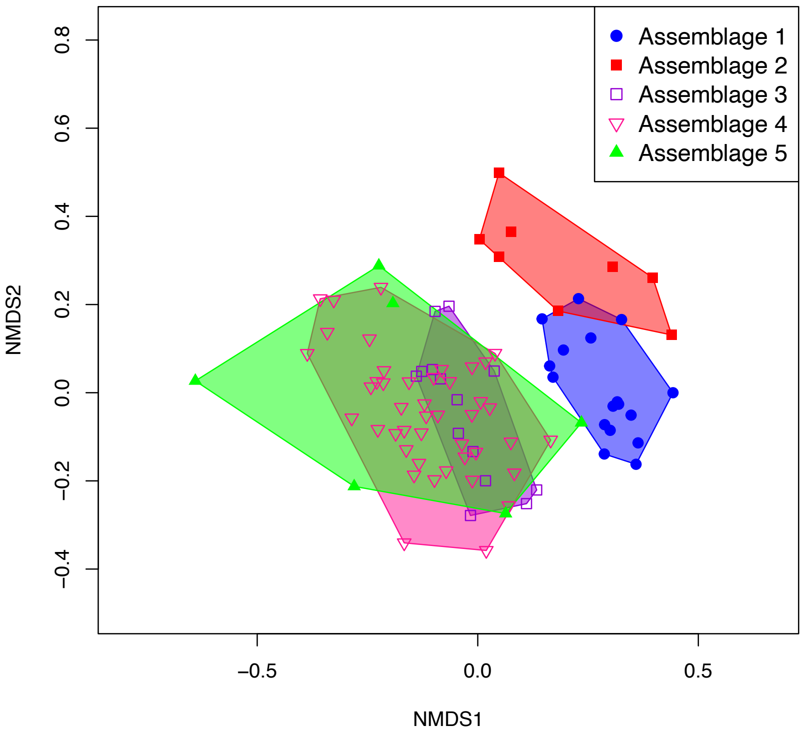

Figure S8. Non-metric multidimensional scaling (NMDS) first and second axis showing (dis)similarity in assemblage composition through time at Site U1553. Assemblage 1 (latest Eocene and earliest Oligocene, 188 to 206 m-CCSF), Assemblage 2 (Early Oligocene interval of *Clausicoccus* acme, 186 to164 m-CCSF), Assemblage 3 (Early Oligocene, 116 to 161), Assemblage 4 (mid- to Late Oligocene, 18 to 113 m-CCSF), Assemblage 5 (Late Oligocene, 6 to 16 m-CCSF). Stress value = 0.13.

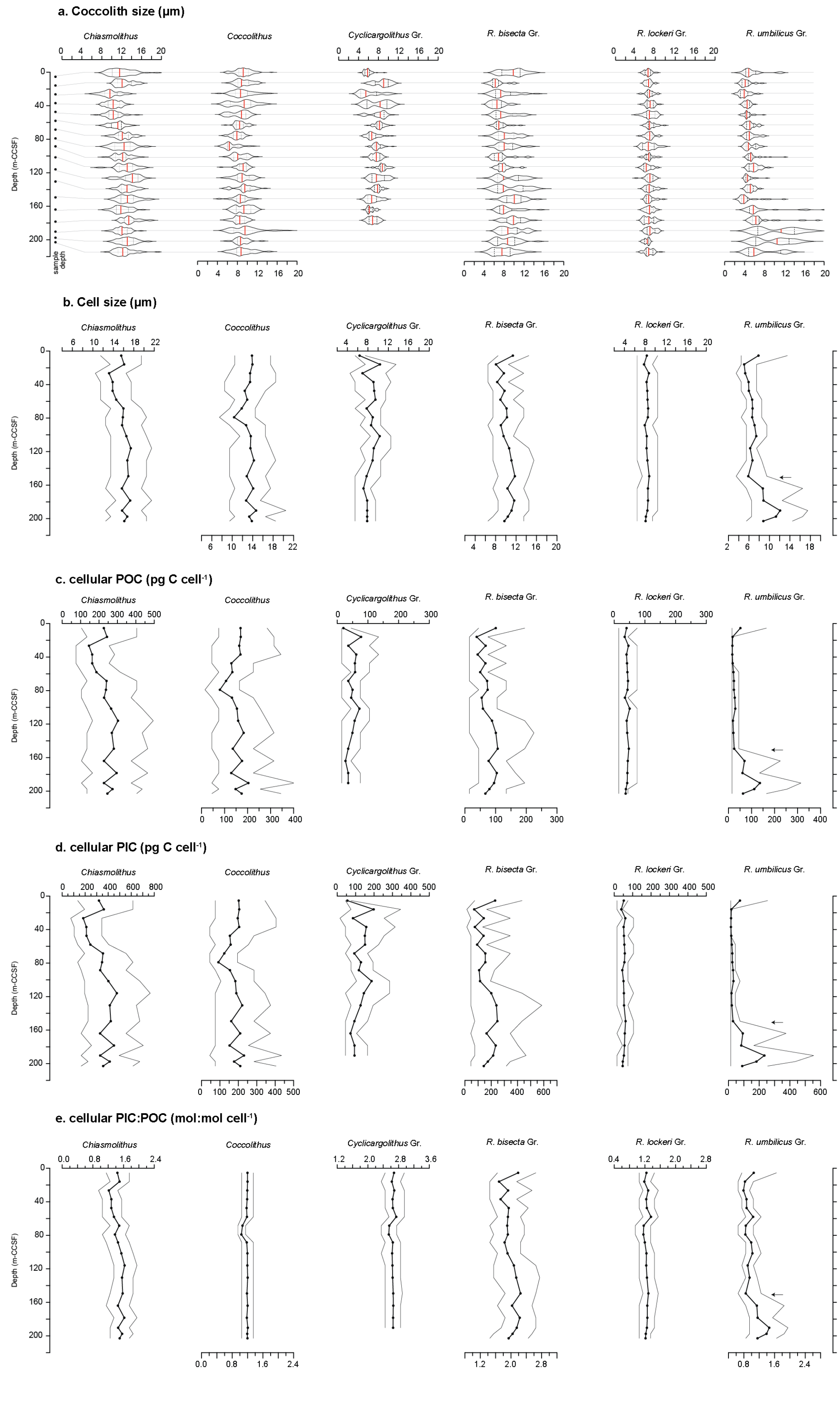
a. Coccolith size (µm)
b. Cell size (µm)
c. cellular POC (pg C cell⁻¹)
d. cellular PIC (pg C cell⁻¹)
e. cellular PIC:POC (mol:mol cell⁻¹)
Chiasmolithus
Coccolithus
Cyclicargolithus Gr.
R. bisecta Gr.
R. lockeri Gr.
R. umbilicus Gr.
Depth (m-CCSF)
sample depth

Figure S9 (previous page). Within-morphogroup (Gr.) modelled cell size and biogeochemical traits (mean, 10th and 90th percentile) through the Oligocene. a. morphogroup measured coccolith length data. b. morphogroup cell size, c. morphogroup cellular particulate organic carbon (POC), d. cellular particulate inorganic carbon (PIC), e. morphogroup cellular PIC:POC. Black arrows on *Reticulofenestra umbilicus* Gr. plots indicate the depth at which T *R. umbilicus* occurs.

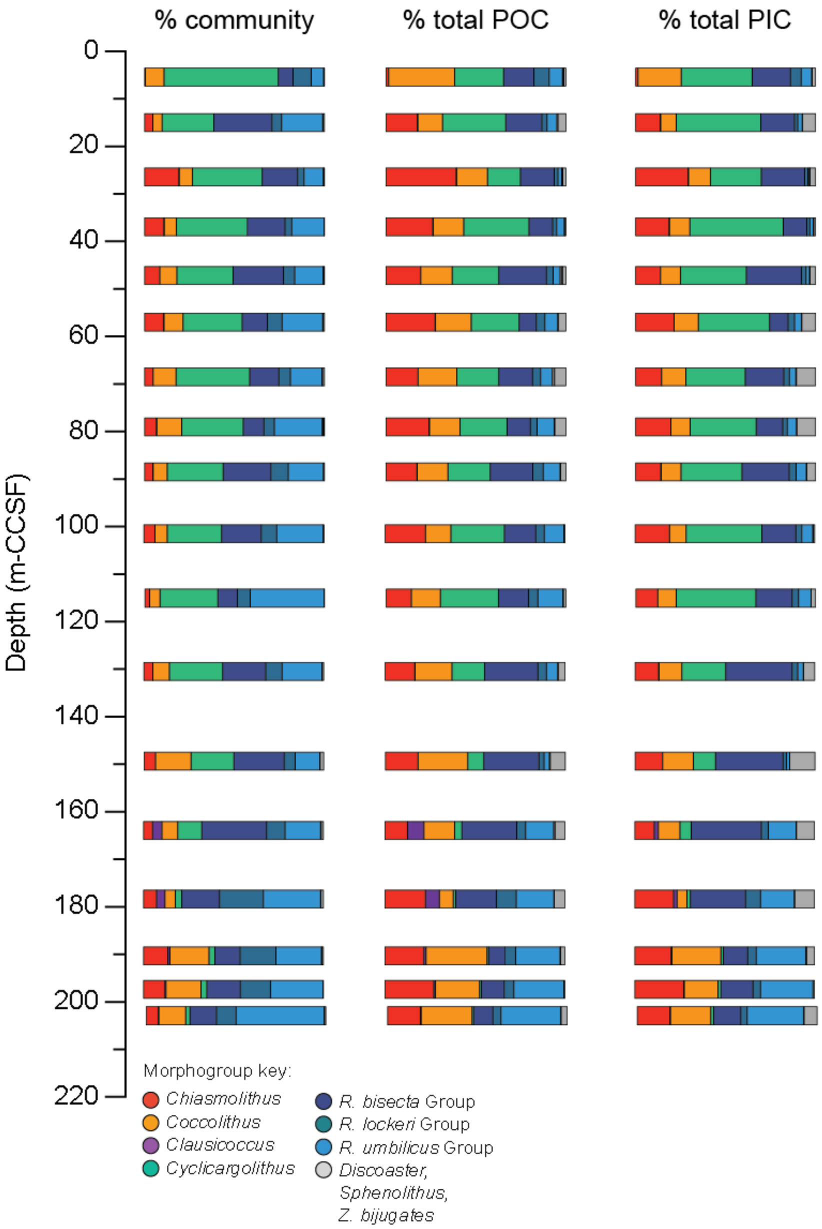

Figure S10. Percentage contribution of each morphogroup to cellular relative abundance, total community POC, and total community PIC through the late Eocene and Oligocene at Site U1553.

Event	Zone/subzone base	Age (Ma)	Reference	Mean Depth (m-CCSF)	Depth source
Unconformity		26.00	Röhl et al. (2022)	4.05	Röhl et al. (2022)
T *Globigerina labiacrassata*	AO4	26.50	Huber & Quillévéré (2005)	17.70	Röhl et al. (2022)
**Early/late Oligocene boundary**		27.82	GTS2012	42.90[a]	This study
Tc *Chiloguembelina cubensis*	AO3	28.10	Huber & Quillévéré (2005)	48.36	Röhl et al. (2022)
T *Subbotina angiporoides*	AO2	29.80	Huber & Quillévéré (2005)	87.92	Röhl et al. (2022)
Tc *Reticulofenestra umbilicus* (> 14 µm)	*Ch. altus*	31.51	Fioroni 2012	150.86	This study
T *Ismolithus recurvus*	*R. daviesii*	32.49	Fioroni 2012	166.06	This study
B *Chiasmolithus altus*	*B. spinosus*	33.31*	Fioroni 2012	186.90[b]	This study
Bi *Clausicoccus subdistichus*	Additional bioevent			186.90	This study
**Eocene/Oligocene Boundary**		33.9	GTS2012	196.04[a]	This study
T *Reticulofenestra oamaruensis*	*R. samodurovi*	33.97	Fioroni 2012	197.12	This study
B *Reticulofenestra oamaruensis*	*R. oamaruensis*	35.54	Fioroni 2012	217.75	Röhl et al. (2022)

Table S1. Eocene and Oligocene bioevents at Site U1553 based on mid-point depths of calcareous nannofossil marker taxa (this study) and depths of other microfossil bioevents (shipboard; Röhl et al. 2022). All datum ages are relative to GTS2012 (Gradstein et al., 2012). T = top, Tc = top common, B = base, Bi = base of acme increase. *denotes a high latitude age for the bioevent, as it is latitudinally diachronous. [a]depth is interpolated based on sedimentation rate between nearest two bioevents. [b]depth is based on first confident identification, as many specimens last distinguishing morphological features.
